# A transcriptome-wide association study implicates specific pre- and post-synaptic abnormalities in schizophrenia

**DOI:** 10.1093/hmg/ddz253

**Published:** 2019-11-06

**Authors:** Lynsey S Hall, Christopher W Medway, Oliver Pain, Antonio F Pardiñas, Elliott G Rees, Valentina Escott-Price, Andrew Pocklington, Nicholas J Bray, Peter A Holmans, James T R Walters, Michael J Owen, Michael C O’Donovan

**Affiliations:** 1 MRC Centre for Neuropsychiatric Genetics and Genomics, Division of Psychological Medicine and Clinical Neurosciences, School of Medicine, Cardiff University, Cardiff CF24 4HQ, UK; 2 Social, Genetic and Developmental Psychiatry Centre, Institute of Psychiatry, Psychology and Neuroscience, King’s College London, London SE5 8AF, UK

## Abstract

Schizophrenia is a complex highly heritable disorder. Genome-wide association studies (GWAS) have identified multiple loci that influence the risk of developing schizophrenia, although the causal variants driving these associations and their impacts on specific genes are largely unknown. We identify a significant correlation between schizophrenia risk and expression at 89 genes in the dorsolateral prefrontal cortex (*P* ≤ 9.43 × 10^−6^), including 20 novel genes. Genes whose expression correlate with schizophrenia were enriched for those involved in abnormal *CNS synaptic transmission* (*P*_FDR_ = 0.02) and *antigen processing and presentation of peptide antigen via MHC class I* (*P*_FDR_ = 0.02). Within the *CNS synaptic transmission* set, we identify individual significant candidate genes to which we assign direction of expression changes in schizophrenia. The findings provide strong candidates for experimentally probing the molecular basis of synaptic pathology in schizophrenia.

## Introduction

Schizophrenia [OMIM: 181500] is a severe psychiatric disorder which typically manifests in late adolescence or early adulthood (1). Genetic studies have shown that schizophrenia is multifactorial but highly heritable. The genetic contribution is polygenic, involving large numbers of risk alleles spanning the full spectrum of possible allele frequencies (2–4). Regarding common variation, the most recent genome-wide association study (GWAS) reported 174 independent association signals representing 145 distinct associated loci (2).

In principle, each genetic locus identified by GWAS provides an opportunity to expose a biological mechanism bridging statistical association and disease. In practice, the imprecision inherent in GWAS makes extracting biological information from these studies challenging. This is based on factors such as extensive linkage disequilibrium, loci spanning multiple genes and regulatory motifs and long-range regulatory mechanisms acting beyond the boundaries of the associated region.

The common variant associations observed in schizophrenia appear to be largely driven by variation affecting gene expression or splicing rather than non-synonymous variation (5, 6). As such, there has been considerable interest in integrating GWAS data with functional annotations defined in appropriate tissues to identify candidate loci that are not detected by GWAS alone. Several methods, collectively described as transcriptome-wide association studies (TWAS) have been developed to achieve this, including summary Mendelian randomization (SMR) (7), PrediXcan (8) and FUSION (9). Here the principle is to derive genomic predictors of gene expression from samples (not necessarily cases) for which both genomic and gene expression data are available, and to use those predictors to impute gene expression in independent samples (e.g. case–control samples) for which genome-wide association data, but not gene expression data, are available.

Several TWAS of schizophrenia have been performed, incorporating a variety of methodologies and source tissues. Applying the FUSION approach to the second GWAS of schizophrenia published by the Psychiatric Genomics Consortium (or PGC2), Gusev and colleagues (10) identified 44 genes for which there was significant evidence for association between schizophrenia and the genetically imputed *cis*-component of gene mRNA expression in adult *postmortem* human brain tissue from the dorsolateral prefrontal cortex (DLPFC). More recently, two studies have used GWAS summary statistics from the larger *CLOZUK + PGC2* schizophrenia meta-analysis (2). Huckins and colleagues (11), using PrediXcan and expression data from 13 brain regions, identified 256 genes for which expression was associated with schizophrenia. The largest number of associations was observed in the DLPFC, with 49 significant genes (outside the MHC), of which 24 were conditionally independent. Gandal and colleagues (12) employed FUSION, incorporating expression data from multiple brain regions across several studies from the PsychENCODE consortium. Gandal and colleagues also employed an SMR approach to further substantiate their findings. They identified 164 genes (out with the MHC). Including the MHC, they identify 193 TWAS significant genes, of which 107 were conditionally independent, and 62 were supported by SMR.

There are large numbers of genes whose mRNA expression in non-brain tissues (e.g. fat, peripheral blood, testis) have also been correlated with schizophrenia (7, 10, 13, 14). While there is clear evidence for cross-tissue overlap in the effects of gene variants on gene expression, unless supported by findings in brain tissue, caution in interpreting those findings in the context of schizophrenia is required.

Here we report a TWAS, and subsequent gene-based analysis, based on the largest published schizophrenia GWAS dataset (2) and publicly available expression data from the CommonMind Consortium study of DLPFC (15). We restricted our analysis to a single tissue source as enrichments for common variant genomic and transcriptome-wide polygenic association signals are effectively restricted to the brain (2, 10); therefore, associations between schizophrenia and heritable gene expression derived from this tissue have the highest plausibility of being biologically relevant to disease pathophysiology. Moreover, as a major aim of our study was to undertake a competitive test-based pathway analysis, the use of a single tissue considerably simplifies this as it minimizes biases towards association with genes expressed in multiple tissues or samples, genes which are also likely to be biased with respect to gene-set membership.

## Results

### Transcriptome-wide association study

**Figure 1:**
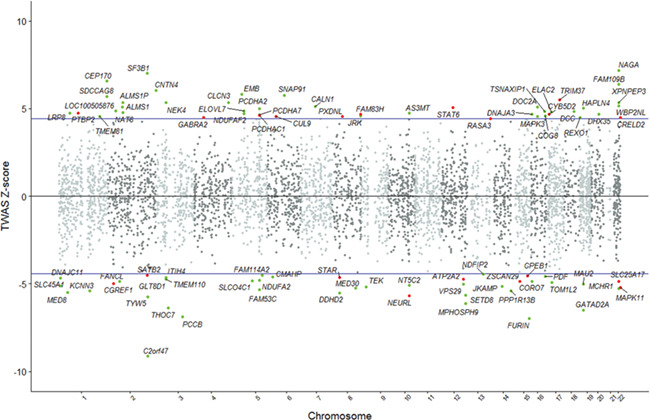
Mirrored Manhattan plot of transcriptome-wide association results. Each point represents a gene, with physical genomic position (chromosome, base-pair) plotted on the *x*-axis and association *Z*-score between gene expression in the dorsolateral prefrontal cortex and schizophrenia plotted on the *y*-axis. Transcriptome-wide-significant associations are labeled and highlighted as green (established) or red (novel) points.

Of the 5301 genes modeled in our data with significant *cis*-heritable expression in the DLPFC, 89 returned a significant TWAS association signal (*P* ≤ 9.43 × 10^−6^). Twenty of these genes are not previously reported as significant associations (Table 1). TWAS test statistics for all genes are shown in Figure 1 and Supplementary Material, Table S1. Schizophrenia liability was not significantly biased towards increased or decreased expression, with increased expression being positively correlated with disease risk at just over half of genes (*N* = 46, sign test *P* = 0.83). Proximal TWAS significant genes (those within a 500-kb window) were aggregated, resulting in 62 discrete loci (Supplementary Material, Table S2). Conditional analyses of each locus identified 68 genes with statistically independent TWAS signals (conditional *P* ≤ 0.05). The TWAS signal could be attributed to a single gene at 56 of these loci (Supplementary Material, Tables S2 and S3).

**Table 1:** Transcriptome-wide association study (TWAS) test statistics for 20 novel significantly associated genes for schizophrenia in the dorsolateral prefrontal cortex (DLPFC). *Top GWAS ID* denotes the SNP in the gene with the largest schizophrenia GWAS *Z*-score, *GWAS Z* is the corresponding *Z*-score for this SNP. *Top eQTL ID* denotes the SNP with the largest DLPFC expression *Z*-score, *Top eQTL Z* is the corresponding *Z*-score for this SNP. *TWAS Z* denotes the gene-level TWAS *Z*-score, *TWAS P* denotes the gene-level TWAS *P* value. Genes which represent signals attributable to a single gene within a TWAS locus are highlighted in bold. Genes which are conditionally independent at loci with multiple conditionally independent signals are highlighted with an asterisk

**Gene**	**Chromosome**	**Gene Start**	**Gene End**	**Top GWAS ID**	**GWAS Z**	**Top eQTL ID**	**Top eQTL Z**	**TWAS Z**	**TWAS P**
***PTBP2***	1	97187174	97280605	rs11165690	4.92	rs17525106	5.96	4.75	2.01E-06
*SATB2*	2	200134222	200335989	rs769949	8.12	rs10931872	3.43	−4.51	6.54E-06
***CGREF1***	2	27322220	27341995	rs4665352	−4.20	rs12474099	−3.20	−4.97	6.64E-07
***GABRA2***	4	46251580	46392056	rs279841	−4.11	rs9291283	−4.52	4.50	6.78E-06
PCDHAC1^*^	5	140306301	140391929	rs991918	4.89	rs753279	−3.40	4.62	3.85E-06
***CUL9***	6	43149921	43192325	rs6938026	−4.98	rs6924555	−8.17	4.54	5.50E-06
***FAM83H***	8	144806102	144815914	rs7465067	4.92	rs13277214	8.39	4.67	2.98E-06
*STAR*	8	38000217	38008600	rs7845911	−6.17	rs2932005	4.45	−4.64	3.50E-06
***PXDNL***	8	52232136	52722005	rs12674620	4.95	rs7008730	3.73	4.55	5.28E-06
NEURL^*^	10	105253734	105352309	rs12413046	−8.51	rs729211	−3.55	−5.68	1.31E-08
*ATP2A2*	12	110719031	110788897	rs4766428	7.61	rs28569834	3.67	−4.73	2.25E-06
***STAT6***	12	57489186	57522883	rs324015	−6.41	rs12307379	6.29	5.05	4.34E-07
***RASA3***	13	114747193	114898095	rs3812831	−5.14	rs1320468	−4.53	4.44	8.89E-06
***ZSCAN29***	15	43650369	43662258	rs1079309	−5.03	rs690276	11.50	−4.85	1.22E-06
***CPEB1***	15	83211950	83316728	rs783540	6.14	rs6603030	5.76	−4.53	5.81E-06
***CYB5D2***	17	4046461	4060995	rs3760471	4.78	rs8067594	4.25	4.69	2.70E-06
***TRIM37***	17	57059999	57184266	rs4968363	−4.57	rs2877926	−4.95	5.51	3.67E-08
*SLC25A17*	22	41165638	41215392	rs926914	6.17	rs80533	−3.95	−4.87	1.09E-06
CRELD2^*^	22	50312282	50321186	rs5770755	4.98	rs910799	3.85	4.49	7.19E-06
MAPK11^*^	22	50702141	50708779	rs5770755	4.98	rs2076139	−4.08	−5.19	2.06E-07

### Correlation with results from summary-based Mendelian randomization

Overall, the effect size estimates from our TWAS analysis were correlated (*R* = 0.71; CI: 0.70–0.73; *P* < 2.2 × 10^−16^, Supplementary Material, Fig. S1) with those obtained using a different methodology (SMR) and an only partially overlapping expression dataset (12). Of the 20 novel genes we report here, only 12 have data reported in the SMR analysis; for that set of genes, the correlation was much stronger (*R* = 0.91; CI: 0.76–0.98; *P* = 1.18 × 10^−5^), with 11 genes significant in the SMR-multi analysis at a threshold allowing for the number of tests (*P* < 0.004) (Supplementary Material, Table S4).

### Correlation with brain-based gene expression in the fetal brain

**Figure 2:**
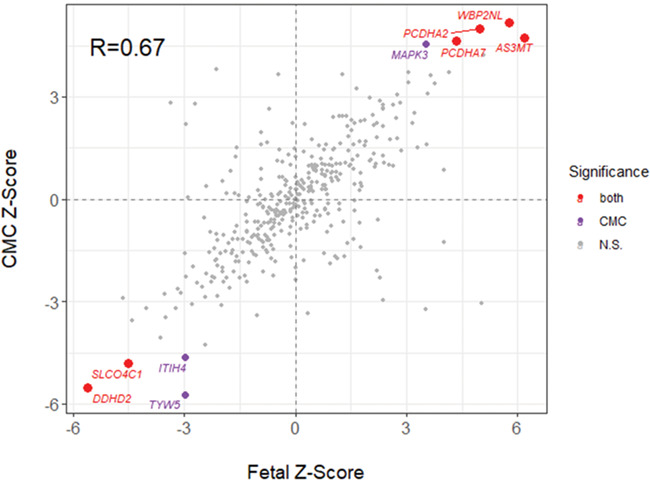
Correlation between transcriptome-wide association study *Z*-scores using expression weights derived from 120 fetal brain homogenate samples and 452 adult dorsolateral prefrontal cortex samples from the CommonMind Consortium (CMC). Points represent genes, with genes significant in both adult and fetal brain tissue highlighted in red, genes significance in adult tissue only highlighted in purple.

The much smaller fetal brain expression dataset yielded fewer testable genes, with only 486 genes testable across the CMC and fetal brain dataset. Across all genes, the effect sizes were correlated *R* = 0.67 (CI: 0.61–0.72; *P* < 2.2 × 10^−16^). Of the nine TWAS-significant genes from the CMC data that are testable in fetal brain, all lie at the very extreme of effect sizes in fetal brain, six are TWAS-significant in fetal brain and all are significant at a threshold allowing for nine genes (Fig. 2).

### Overlap with putative GWAS loci

Thirty-five of the 62 TWAS significant loci, incorporating 56 TWAS-significant genes, could be mapped to within 500 kb of genome-wide-significant loci reported in the schizophrenia GWAS upon which this TWAS was based (2) (Supplementary Material, Table S5). It was possible to distinguish a single significant TWAS gene at 32 of these loci, after conditioning on other TWAS signals at the locus (Supplementary Material, Table S6). At 28 of these, conditioning on the imputed expression of the candidate gene nominated by TWAS was sufficient to reduce sentinel SNP associations to below genome-wide significance (*P* ≥ 5 × 10^−8^) without unmasking a secondary signal, suggesting that these candidates potentially fully account for the association signals at the locus. Conditional regional association plots for the 32 loci where a single gene was driving the signal are shown in Supplementary Material, Figs S2–S33. Twenty-seven TWAS-significant loci, comprising 33 significant TWAS genes, fell outside established (genome-wide-significant) schizophrenia risk loci (+/−500 kb) as defined by Pardiñas *et al*. (2). Of these, 24 loci had a signal attributable to one gene (Supplementary Material, Table S6). TWAS-significant associations at loci not (yet) implicated in GWAS can arise when multiple variants influence the phenotype through changes in expression, or through the reduced multiple testing burden of gene-based analysis (10, 14).

### Gene-set analysis

Of the 2390 gene-sets which included at least 10 *cis*-heritable genes, significant enrichment after correction for multiple testing (*P*_FDR_ ≤ 0.05) was observed for two gene sets: *abnormal CNS synaptic transmission* (16) (*P*_FDR_ = 0.02) and *antigen processing and presentation of peptide antigen via MHC class I* (GO:0002474; *P*_FDR_ = 0.02) (Table 2, and Supplementary Material, Table S7 for all gene sets). *Abnormal CNS synaptic transmission* contained six TWAS-significant genes (Supplementary Material, Table S8, and discussed in the following), but GO:0002474 did not contain any TWAS-significant genes (Supplementary Material, Table S9).

**Table 2:** Gene-set analysis test statistics for gene sets which survived multiple testing correction. *Beta (SE)* denotes the regression coefficient and its corresponding standard error. *N cis-h^2^ genes in set* denotes the number of *cis*-heritable genes in the gene-set used in analysis; *N genes in set* denotes the total number of genes in gene-set; *P value* denotes the association *P* value; *P_FDR_* denotes the *P* value corrected using a false discovery rate of 0.05

**Gene set**	**Beta (SE)**	***N cis*-h** ^**2**^ **genes in set**	***N* genes** **in set**	***P* value**	***P***_**FDR**_
Antigen processing and presentation of peptide antigen via MHC class I (GO:0002474)	1.03 (0.24)	25	98	1.07 × 10^−5^	0.023
Abnormal CNS synaptic transmission	0.58 (0.14)	84	392	1.90 × 10^−5^	0.023

To illustrate that gene-set enrichment tests based on well-powered GWAS and brain-expressed genes do not generate significant associations to plausible CNS-related gene sets simply by virtue of tissue of eQTL origin, we used the same procedures to perform an identical gene-set analysis using summary statistics from a well-powered GWAS of a non-CNS, but highly heritable and polygenic, phenotype, namely height (17). Despite yielding many more (*n* = 287) TWAS-significant genes, no gene sets were significant using our procedures (minimum *P*_FDR_ = 0.83).

None of the gene sets which were significant in the MAGMA (18) analysis of the contributing GWAS (2) were associated (after correction) in the current analysis, with only *abnormal long term potentiation* (MP:0002207) and genes classed as *Loss of Function Intolerant* showing nominal significance (*P* = 2.38 × 10^−4^ and 0.014, respectively) (Supplementary Material, Table S7). In particular, the gene-set *FMRP targets* were not even nominally significant despite strong evidence for association in that study (2). One difference between TWAS- and GWAS-MAGMA analyses is that the former includes only those genes detected to be significantly *cis*-heritable, while the latter includes all genes with SNPs mapping to physical boundaries surrounding the gene. Thus, TWAS only includes a fraction of the genes that are included in the GWAS-MAGMA (e.g. here, only 19%). As the gene-set tests are designed to be competitive (i.e. the association statistics in the specified set are contrasted against a background set of all testable genes), this affects both the gene sets and the background set.

First, we postulated that the *cis*-heritable subset of FMRP target genes included in the TWAS might be selectively depleted of genes that confer the association signal to the gene set in the GWAS. This hypothesis arose from the observation that FMRP targets are underrepresented for *cis*-heritability relative to other brain-expressed genes, comprising only 3.2% of genes with *cis*-heritable expression in DLPFC but 8.9% of genes expressed in brain-tissue (19) which showed no evidence of *cis*-heritability (*χ*^2^ test of differences between the proportion *P* = 3.84 × 10^−37^). However, GWAS-based analysis, implemented in MAGMA (18), of the genes with (165 genes) and without (652 genes) *cis*-heritable expression did not support this hypothesis (*cis*-heritable sub-set; *B* = 0.31, SE = 0.09, *P*_FDR_ = 2.2 × 10^−4^: non-*cis*-heritable set; *B* = 0.22, SE = 0.05, *P*_FDR_ = 5.99 × 10^−7^), although the non-*cis*-heritable set was more significant based on the greater number of genes included. Second, we tested the effect of restricting the background of the MAGMA analysis to include only genes with the DLPFC *cis*-heritable genes used in the TWAS. This reduced the significance of GWAS-MAGMA association by six orders of magnitude (*B* = 0.36, SE = 0.10, *P*_FDR_ = 0.03) relative to the standard test (*B* = 0.25, SE = 0.04, *P*_FDR_ = 6.01 × 10^−8^). Third, we determined the overlap of SNP mapping to FMRP targets in the MAGMA and TWAS analysis. As expected, many fewer SNPs entered the TWAS than the GWAS-MAGMA analysis (78 260 vs. 447 742), and of those that did, only 18 112 (i.e. 23% of TWAS SNPs, 4% of MAGMA SNPs) were common to both analyses. Both the substantial reduction in the number of SNPs and the poor overlap between MAGMA and TWAS SNPs credibly explain much of the difference between the two analyses.

We conclude that the discrepancy between TWAS and GWAS is not explained by selective depletion of *cis*-heritable genes for GWAS association; rather, it is likely a combination of lower power based on inclusion of fewer genes and SNPs, changes in the attribution of SNPs to genes and attenuation of signal based on a more restricted background set. Interestingly, FMRP targets were significantly associated in the PrediXcan study (12) study, possibly for related reasons—given this method does not so markedly constrain the number of genes to those with demonstrable *cis*-heritable genes expression.

## Discussion

### Summary of findings

In the current study, we identified significant correlations between *cis*-expression in the DLPFC and schizophrenia risk at 89 genes, of which 20 are novel. We determined that disease risk was correlated to the *cis*-expression of a single gene at 56 loci, 32 of which have previously been implicated by schizophrenia GWAS. Of these 56 genes, 12 are novel. Prioritizing putative causal candidate genes and predicted directions of effect at GWAS loci are important steps towards facilitating functional experimentation.

To validate our findings, we compared our results to those obtained using a different methodological approach (SMR) and with results from a TWAS of independent brain tissue from fetal samples. Our data showed a strong correlation and, where our specific novel associated genes were testable, significant support from both additional analyses. Thus, our results are reproducible at both the methodological and source tissue levels.

The high correlation between the results of the TWAS based on fetal and adult gene expression suggests risk alleles for schizophrenia that influence gene expression often do so in a relatively (developmentally) non-specific way, but this does not imply the timing of critical pathogenic events is developmentally non-specific. Moreover, given the limited sample size of the samples, this may only apply to *cis*-acting alleles that have very large effects on fetal brain (and hence are detectable in the small fetal brain sample). Better-powered samples that capture more of the *cis*-heritable gene expression are needed.

Gene-set analysis of the schizophrenia TWAS results implicated two gene sets after conservative correction. The gene set GO:0002474 is intriguing given the well-established association between schizophrenia and common variation at the MHC class l, and the long-standing hypotheses relating immunity and inflammation to the disorder. However, tempting as it is to link these observations and hypotheses, functional interpretation of this finding is limited by the absence of any individually associated genes in the set. Moreover, those genes showing the strongest evidence of association have a diverse range of proteolytic functions unrelated to antigen processing, for example *PSMA4* encodes a component of the proteasome complex and is involved in degradation of almost all intracellular proteins. Thus, we suggest that speculation about the significance of that finding awaits more robust implication of individual genes. In contrast, the gene-set *abnormal CNS synaptic transmission* contains six TWAS-significant genes and implicates processes relevant to glutamatergic and GABAergic transmission. These processes have been implicated by rare variant studies of schizophrenia (16, 20–23), but the findings from common variant studies have, to date, been equivocal. In the context of being individually associated, and their membership of biological processes that are implicated in the disorder, these TWAS-significant genes represent a particularly interesting subset of candidates for generating biological hypotheses and are discussed further in the following.

### Candidates within the abnormal CNS synaptic transmission set

*GABRA2*, *CLCN3*, *DOC2A*, *MAPK3*, and *LRP8* are significantly upregulated (in cases) while *CPEB1* is significantly downregulated (Supplementary Material, Table S8). Both *GABRA2* and *CPEB1* represent novel associations. *GABRA2* encodes the gamma-aminobutyric acid type A (GABA_A_) receptor alpha-2 subunit which, with other subunits, form GABA_A_ receptors—the main inhibitory receptors in the CNS. A role for altered GABAergic function in schizophrenia has been proposed (21–24), largely on the basis of imaging studies, animal models of putative intermediate phenotypes and *postmortem* expression studies (25). In the latter, consistent with our results, elevated RNA expression (12) and immunoreactivity (26) for *GABRA2* have been observed in the DLPFC from individuals with schizophrenia. Currently, the *postmortem* and *in vivo* imaging data are not decisive in pointing to a primary excess or decrease of GABAergic signaling at glutamatergic neurons in schizophrenia (27). Our finding that elevated *cis*-expression of *GABRA2* is associated with increased risk of schizophrenia suggests that the change contributes to pathogenic processes rather than being simply compensatory (see also *CLCN3* in the following) and suggests the possibility that targeting treatments at antagonism at this receptor complex might be more appropriate than agonism, the focus to date. At a wider level, this finding adds further genetic support for the GABAergic hypothesis of schizophrenia and complements a recent study which reported enrichment for components of GABA_A_ receptor complexes in CNVs in people with schizophrenia (16).

*CLCN3* is involved in regulating neurotransmitter vesicle turnover at both excitatory glutamatergic and inhibitory GABAergic synapses, can be pre- or postsynaptic and is thought to be an important regulator of synaptic plasticity (28, 29). Neurotransmitter release at inhibitory GABAergic synapses is reduced in CLCN3-null mice while glutamate release is increased (28, 29); our finding, and that of another study based on the SMR approach (15), of increased expression of this gene might suggest the reverse effect (reduced glutamate, increased GABA) in schizophrenia consistent with the aberrance of both excitatory and inhibitory signaling in schizophrenia.

*DOC2A* and *MAPK3*, encoding double C2 domain alpha and mitogen-activated protein kinase 3, respectively, map to 16p11.2, duplication of which is associated with schizophrenia (30). In the present study, conditional analysis suggests expression of both genes contributes to the association signal at this locus, the conditional evidence being much stronger for expression of *DOC2A* (conditional on *MAPK3*, *P* = 3.5 × 10^−7^) than *MAPK3* (conditional on *DOC2A*, *P* = 0.014). Reduction in *DOC2A* expression results in a reduction in spontaneous glutamate release from hippocampal neurons in culture and increases glutamatergic synaptic efficiency and synaptic efficacy (31), suggesting the possibility that the increased *cis*-expression predicted by the TWAS might result in hypofunction at glutamatergic synapses. In addition to previously discussed findings implicating *MAPK3* in schizophrenia (10), we note that *MAPK3* RNA expression is also found to be increased in the *postmortem* frontal cortex from people with schizophrenia, in contrast with controls, in the study of Gandal and colleagues (12).

*CPEB1* (reduced *cis*-expression) encoding cytoplasmic polyadenylation element-binding protein 1, is expressed in dendrites where it facilitates activity-dependent mRNA translation (32, 33) and memory consolidation (34). It has been reported to bind, and act antagonistically to, FMRP which is a translational repressor (34), targets of which have been repeatedly linked to schizophrenia (2, 19, 35). Moreover, a recent study (36) has shown that CPEB4 protein is an important regulator of a large number of autism-associated genes. Our study suggests the possibility that *CPEB1* might similarly regulate genes that play a role in schizophrenia, possibly by interaction with FMRP.

*LRP8*, which encodes LDL receptor-related protein 8, is widely expressed, but is particularly highly expressed in the developing fetal brain (37). *LRP8* is a component of the NMDA receptor complex (NMDA-R) (38), which has repeatedly implicated in schizophrenia (16, 35, 39). It is also the major receptor, and effector, of the actions of reelin as a modulator of plasticity, memory and learning in the adult brain (40). It should be noted that *LRP8* has non-synaptic functions of credible relevance to schizophrenia, including mediating reelin-related functions on neuronal migration, neurogenesis and neuronal differentiation (41). However, these processes are not implicated by the gene-set analysis.

### Comparison with existing TWAS literature

Of the 89 TWAS significant genes, 11 overlap with genes reported as significant by Huckins *et al*. (11), and 45 overlap with those reported as significant Gandal *et al*. (12). The novel genes presented in each study, despite using the same GWAS summary statistics (2), is to be expected based on the analytical differences between these studies. Huckins *et al*. used a cross-brain-tissue approach implemented in PrediXcan, which has different inclusion criteria and a different modeling approach (using exclusively ‘enet’ regression method, where FUSION employs four regression frameworks).

Gandal *et al.*, as in the current manuscript, employed a FUSION-based approach, but utilized brain weights derived from the PsychENCODE consortium (*N* = 1321), which combine multiple brain regions from several studies. This increased statistical power in the expression dataset results in a starting set of 14 750 significantly *cis*-h^2^ genes for analysis.

There are strengths and limitations to the various approaches. Taking a more inclusive approach to expression source tissue may well increase power to detect larger numbers of genes, although based on substantial regional differences in gene expression, the opposite is sometimes true as shown by Collado-Torres *et al*. (42). In contrast, restricting the analysis to a single tissue considerably simplifies pathway-based competitive tests, as it minimizes biases towards finding association with genes expressed in multiple tissues or samples, genes which are not likely to be random with respect to gene-set membership, violating the assumptions of the competitive test.

## Conclusion

In summary, we have undertaken a single tissue TWAS exploiting the largest available published schizophrenia GWAS dataset. We identify 20 novel genes whose expression correlates with schizophrenia, six of which implicate processes of high biological plausibility related to synaptic function. Our findings are congruent with those employing different methodologies and with expression in the fetal brain. These candidate genes, to which we assign predicted directions of effect, could facilitate experimental studies geared towards a better mechanistic understanding of schizophrenia pathogenesis.

## Materials and Methods

### Datasets

We performed TWAS using GWAS summary statistics from the *CLOZUK + PGC2* schizophrenia meta-analysis (2) (40 675 cases, 64 643 controls). Summary statistics were filtered prior to use in FUSION using the munge_sumstats.py (v2.7.13) script, distributed as part of the LD score regression package (URLs). Given its localized pattern of long-range and complex LD, variants within the extended MHC region (chr6:28477797–33 448 354) were excluded, leaving 6 414 705 polymorphisms.

Pre-computed expression weights for 5420 genes with *cis*-heritable expression in the DLPFC were obtained from the FUSION website (URLs). Weights were calculated using bulk RNA sequence and genotype data from schizophrenia cases, bipolar cases and control subjects as part of the CommonMind Consortium(15) (*N* = 452). Gene-expression and genotype data from the CMC were processed prior to weight derivation using the GTEx Consortium guidelines for eQTL analysis of RNA-Seq data, described in detail elsewhere (10, 15).

Reference haplotypes from the 1000 Genomes European subpopulation were also obtained from the FUSION website.

### Transcriptome-wide association study

TWAS was performed in FUSION (9), using the *FUSION.assoc.test.R* script with default parameters over all autosomes (URLs). Multiple-testing correction was applied using the Bonferroni method. Where multiple proximal (+/− 500 kb) genes reported a significant TWAS association, statistically independent signals were discriminated via conditional analysis using the *FUSION.post_process.R* script with default parameters. Genes which remained significant after conditional analysis and locus-wide Bonferroni correction (conditional *P* ≤ 0.05/2*Ngenes, to account for pairwise testing) were considered statistically independent.

### Correlation with results from summary-based Mendelian randomization

To assess the robustness of our findings to a different analytical method and an only partially overlapping expression dataset, we performed a Pearson’s rank correlation test between the effect size estimates from our TWAS analysis and those derived from SMR as implemented in (12), for the subset (*N* = 4327) of genes present in both datasets.

### Correlation with brain-based gene expression in the fetal brain

To assess the robustness of our results to tissue source, and also the relevance of our findings with respect to gene regulation in early development, we repeated the TWAS analysis using expression weights for 1280 genes with *cis*-heritable expression in fetal whole brain tissue. Weights were derived, using the FUSION pipeline, from whole transcriptome RNA sequencing of brain homogenate samples and genome-wide genotyping of 120 human fetuses aged 12–19 post-conception weeks, the processing of which is described in detail elsewhere (43). Multiple-testing correction was applied using the Bonferroni method. TWAS *Z*-scores from genes analyzed in both adult and fetal brain tissue (*N* = 386) were subjected to a Pearson’s rank correlation test.

### Overlap with putative GWAS loci

Significant TWAS loci were mapped to the genomic coordinates for 145 schizophrenia-associated loci from the meta-analysis (2). At each locus containing a TWAS signal which could be attributed to a single gene, locus-wide GWAS statistics were conditioned on imputed gene expression using the *FUSION.post_process.R* script. The change in *P* value of the most significant polymorphism within the locus, before and after conditional analysis, was used to evaluate the effect of imputed expression on phenotypic (schizophrenia) association.

### Gene-set enrichment analysis

A competitive gene-set enrichment analysis was performed using a parallel approach to MAGMA (44, URLs), implementing a mixed linear regression framework, fitted using the lme4qtl package (45), in R (46). The model fitted probit-transformed TWAS *Z*-score as the dependent variable and gene-set membership as a linear predictor, covarying for *gene-length* and *number of SNPs in the gene* as fixed effects, and a correlation matrix of gene expression as a random effect to account for non-independent association arising both from linkage disequilibrium and close physical distance between genes. The correlation matrix was computed by imputing predicted gene expression values, using the same CommonMind Consortium expression weights for genes (*N* = 5301) as the primary analysis, into the 1000 Genomes European subpopulation (*N* = 489). The correlation between genes that were more than 5 Mb apart, on separate chromosomes or had a linkage disequilibrium *R*^2^ < 0.0001 were set to zero. This was carried out for 2390 gene sets which contained 10 or more *cis*-heritable genes, representing a TWAS-informative subset of a larger data-driven, comprehensive annotation of gene sets from multiple databases (*N* = 6677), as per Pardiñas *et al*. (2). In brief, this analysis comprised custom annotated CNS gene sets (16); a gene set of genes intolerant to loss-of-function mutations (20); gene sets from Gene Ontology database release 01/02/2016 (47, 48); the fourth ontology level of the MGI database version 6(49); REACTOME version 55 (50); KEGG release 04/2015 (51); and OMIM release 01/02/2016 (52). Further details about these gene sets can be found here (2, 16). Gene sets were corrected for multiple testing using a 5% Benjamini & Hochberg false discovery rate (FDR).

## URLs

Schizophrenia GWAS summary statistics:

https://walters. psycm.cf.ac.uk/.

FUSION scripts, SNP-weights for CMC and 1000 genomes reference panel plink files: http://gusevlab.org/projects/fusion/

TWAS-based gene-set enrichment analysis software:

https://github.com/opain/TWAS-GSEA

LD Score Regression:

https://github.com/bulik/ldsc

BRAINSPAN transcriptomes:

http://www.brainspan.org/static/download.html

## Supplementary Material

Suppl_data_ddz253
