# Supplementary material for: A transcriptome-wide association study implicates specific pre- and post-synaptic abnormalities in schizophrenia
Source: Hum Mol Genet. 2019 Nov 6;29(1):159–67. doi: 10.1093/hmg/ddz253 (PMC7416679; doi:10.1093/hmg/ddz253)
Supplement: Suppl_data_ddz253 [file suppl_data_ddz253.zip › Hall_et_al_Supplementary_Figure_Legends.docx]

**Hall *et al* A Transcriptome-Wide Association Study implicates specific pre- and post-synaptic abnormalities in Schizophrenia**

**Legends for Supplementary Figures 1 - 33**

**Supplementary Figure 1:** Correlation plot of TWAS Z-scores from the current study (PGC2+CLOZUK) against SMR Betas from the Gandal *et al* study (PsychENCODE; PMID: 30545856) demonstrating cross-methodological consistency in direction of effect.

**Supplementary Figures 2-33:** Conditional regional association plots for the 32 loci where a single gene was driving the signal. The top panel shows all the genes in the locus, with marginally associated TWAS genes in blue, and conditionally significant associated TWAS genes in green. The top panel shows a Manhattan plot of the contributing GWAS data. Each dot represents the GWAS -log10(P-value) for a SNP in the locus before (grey) and after (blue) conditioning on gene expression of the conditionally independent (green) genes).

**Supplementary Figure 2:** Regional association plot for *SLC45A1*.

**Supplementary Figure 3:** Regional association plot for *MED8*.

**Supplementary Figure 4:** Regional association plot for *CEP170*.

**Supplementary Figure 5:** Regional association plot for *FANCL*.

**Supplementary Figure 6:** Regional association plot for *ALMS1P*.

**Supplementary Figure 7:** Regional association plot for *SF3B1*.

**Supplementary Figure 8:** Regional association plot for *C2orf47*.

**Supplementary Figure 9:** Regional association plot for *CNTN4*.

**Supplementary Figure 10:** Regional association plot for *NEK4*.

**Supplementary Figure 11:** Regional association plot for *THOC7*.

**Supplementary Figure 12:** Regional association plot for *PCCB*.

**Supplementary Figure 13:** Regional association plot for *CLCN3*.

**Supplementary Figure 14:** Regional association plot for *EMB*.

**Supplementary Figure 15:** Regional association plot for *FAM53C*.

**Supplementary Figure 16:** Regional association plot for *CMAHP*.

**Supplementary Figure 17:** Regional association plot for *SNAP91*.

**Supplementary Figure 18:** Regional association plot for *DDHD2*.

**Supplementary Figure 19:** Regional association plot for *JRK*.

**Supplementary Figure 20:** Regional association plot for *STAT6*.

**Supplementary Figure 21:** Regional association plot for *VPS29*.

**Supplementary Figure 22:** Regional association plot for *MPHOSPH9*.

**Supplementary Figure 23:** Regional association plot for *NDFIP2*.

**Supplementary Figure 24:** Regional association plot for *JKAMP*.

**Supplementary Figure 25:** Regional association plot for *PPP1R13B*.

**Supplementary Figure 26:** Regional association plot for *CPEB1*.

**Supplementary Figure 27:** Regional association plot for *FURIN*.

**Supplementary Figure 28:** Regional association plot for *TSNAXIP1*.

**Supplementary Figure 29:** Regional association plot for *TOM1L2*.

**Supplementary Figure 30:** Regional association plot for *GATAD2A*.

**Supplementary Figure 31:** Regional association plot for *DHX35*.

**Supplementary Figure 32:** Regional association plot for *XPNPEP3*.

**Supplementary Figure 33:** Regional association plot for *NAGA*.
