# Supplementary material for: A transcriptome-wide association study implicates specific pre- and post-synaptic abnormalities in schizophrenia
Source: Hum Mol Genet. 2019 Nov 6;29(1):159–67. doi: 10.1093/hmg/ddz253 (PMC7416679; doi:10.1093/hmg/ddz253)
Supplement: Suppl_data_ddz253 [file suppl_data_ddz253.zip › Supplementary_Figures_1_to_33.pdf]

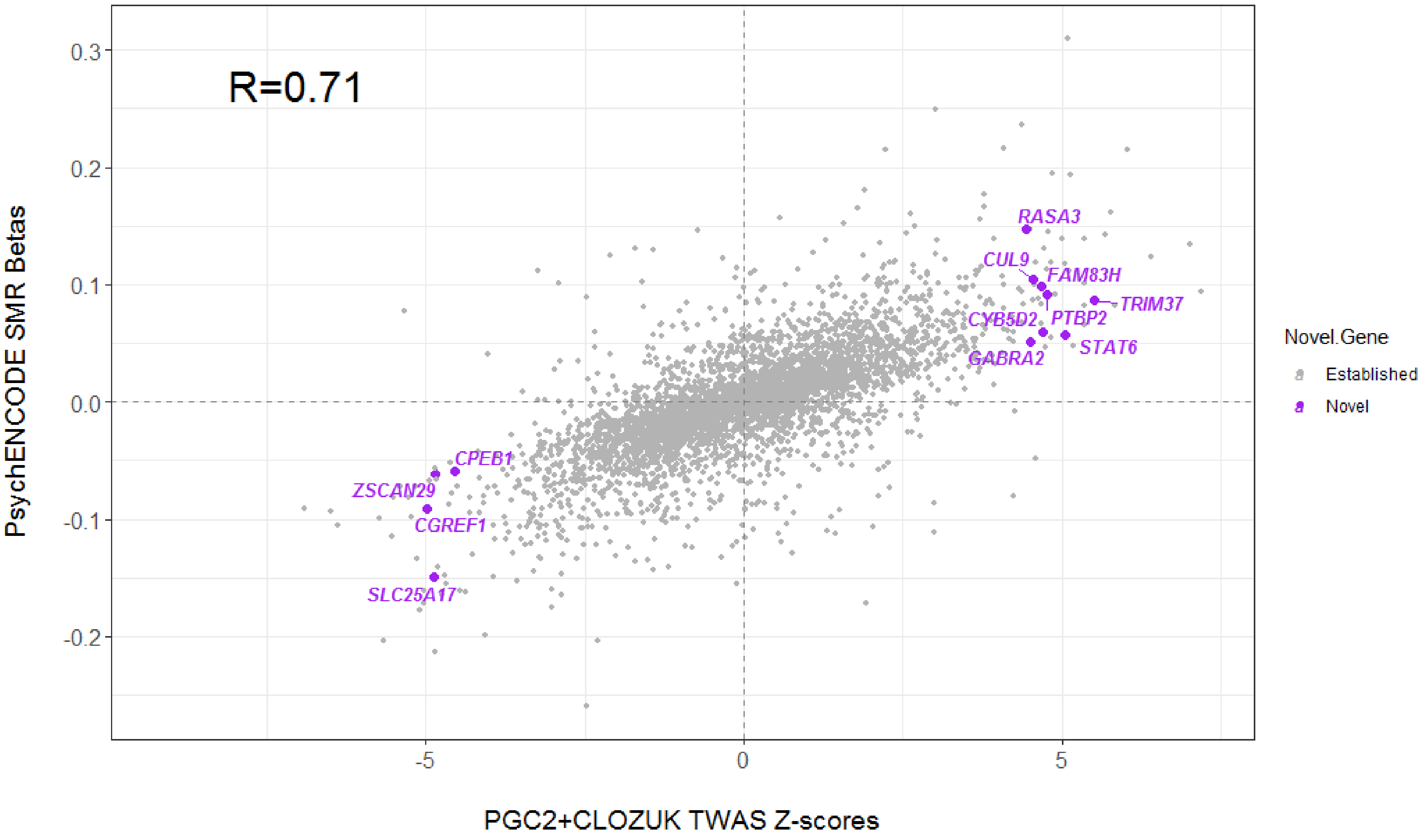

R=0.71
PsychENCODE SMR Betas
PGC2+CLOZUK TWAS Z-scores
0.3
0.2
0.1
0.0
-0.1
-0.2
-5
0
5
RASA3
CUL9
FAM83H
TRIM37
CYB5D2
PTBP2
STAT6
GABRA2
CPEB1
ZSCAN29
CGREF1
SLC25A17
Novel.Gene
Established
Novel

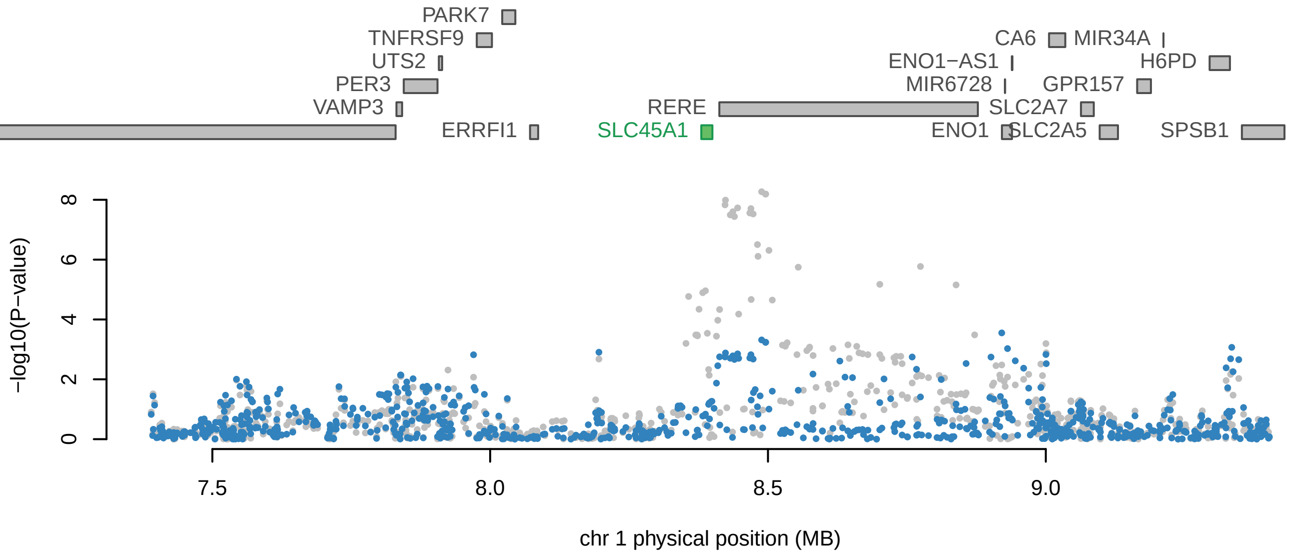

PARK7
TNFRSF9
UTS2
PER3
VAMP3
ERRFI1
SLC45A1
RERE
CA6
MIR34A
ENO1−AS1
H6PD
MIR6728
GPR157
SLC2A7
ENO1
SLC2A5
SPSB1
−log10(P−value)
0
2
4
6
8
7.5
8.0
8.5
9.0
chr 1 physical position (MB)

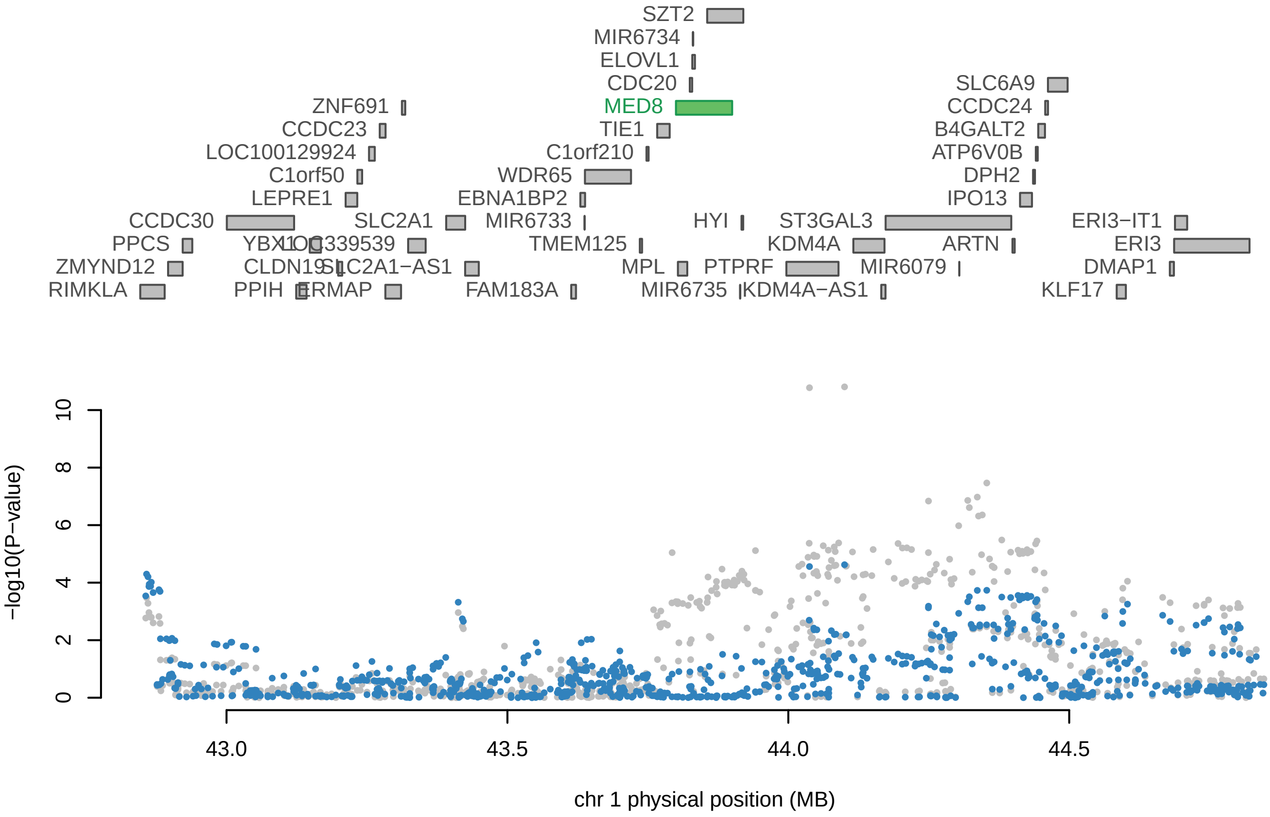
SZT2
MIR6734
ELOVL1
CDC20
MED8
TIE1
C1orf210
WDR65
EBNA1BP2
MIR6733
TMEM125
MPL
MIR6735
FAM183A
ZNF691
CCDC23
LOC100129924
C1orf50
LEPRE1
CCDC30
SLC2A1
PPCS
YBX1
LOC339539
ZMYND12
CLDN19
SLC2A1−AS1
RIMKLA
PPIH
ERMAP
HYI
ST3GAL3
KDM4A
PTPRF
KDM4A−AS1
MIR6079
ARTN
SLC6A9
CCDC24
B4GALT2
ATP6V0B
DPH2
IPO13
ERI3−IT1
ERI3
DMAP1
KLF17
−log10(P−value)
0
2
4
6
8
10
43.0
43.5
44.0
44.5
chr 1 physical position (MB)

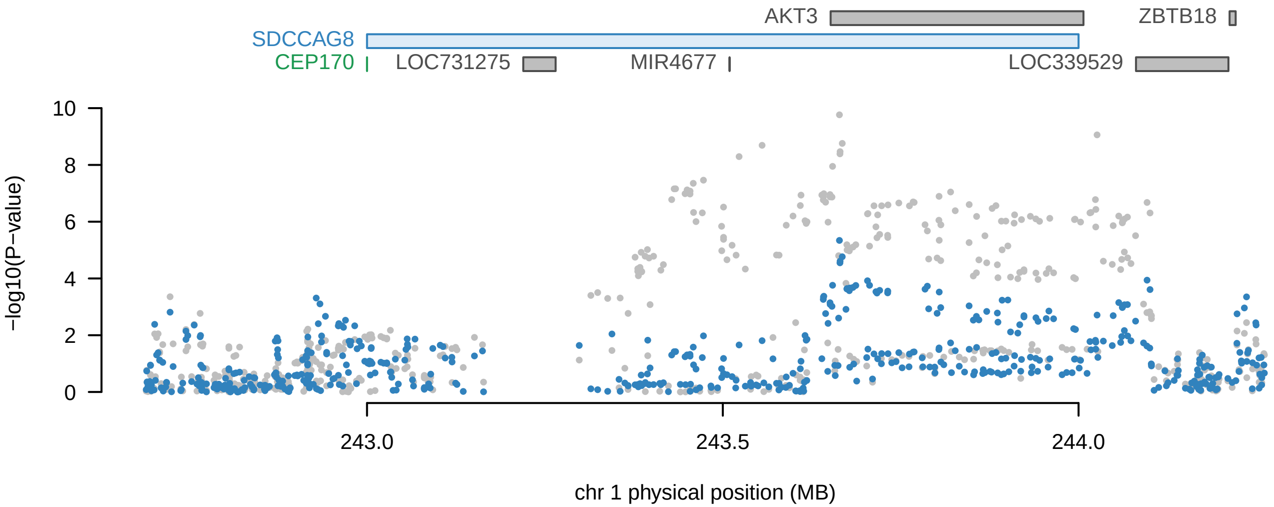
AKT3
ZBTB18
SDCCAG8
CEP170
LOC731275
MIR4677
LOC339529
10
8
6
4
2
0
−log10(P−value)
243.0
243.5
244.0
chr 1 physical position (MB)

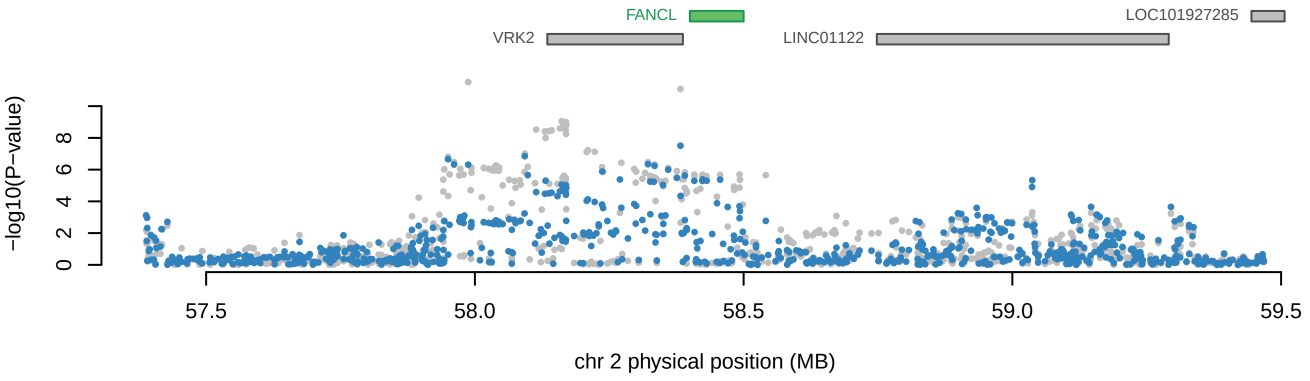
FANCL
LOC101927285
VRK2
LINC01122
−log10(P−value)
0
2
4
6
8
57.5
58.0
58.5
59.0
59.5
chr 2 physical position (MB)

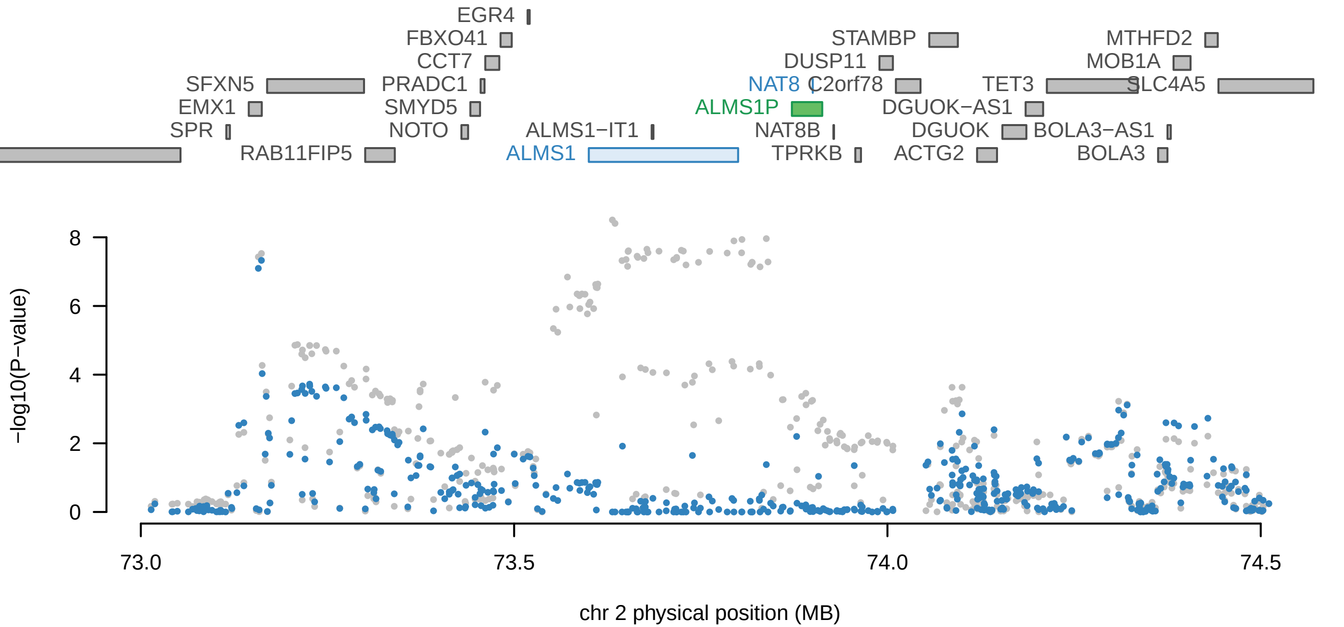
EGR4
FBXO41
CCT7
SFXN5
PRADC1
EMX1
SMYD5
SPR
NOTO
RAB11FIP5
ALMS1−IT1
ALMS1
STAMBP
DUSP11
NAT8
C2orf78
ALMS1P
NAT8B
TPRKB
MTHFD2
MOB1A
TET3
SLC4A5
DGUOK−AS1
DGUOK
BOLA3−AS1
ACTG2
BOLA3
−log10(P−value)
0
2
4
6
8
73.0
73.5
74.0
74.5
chr 2 physical position (MB)

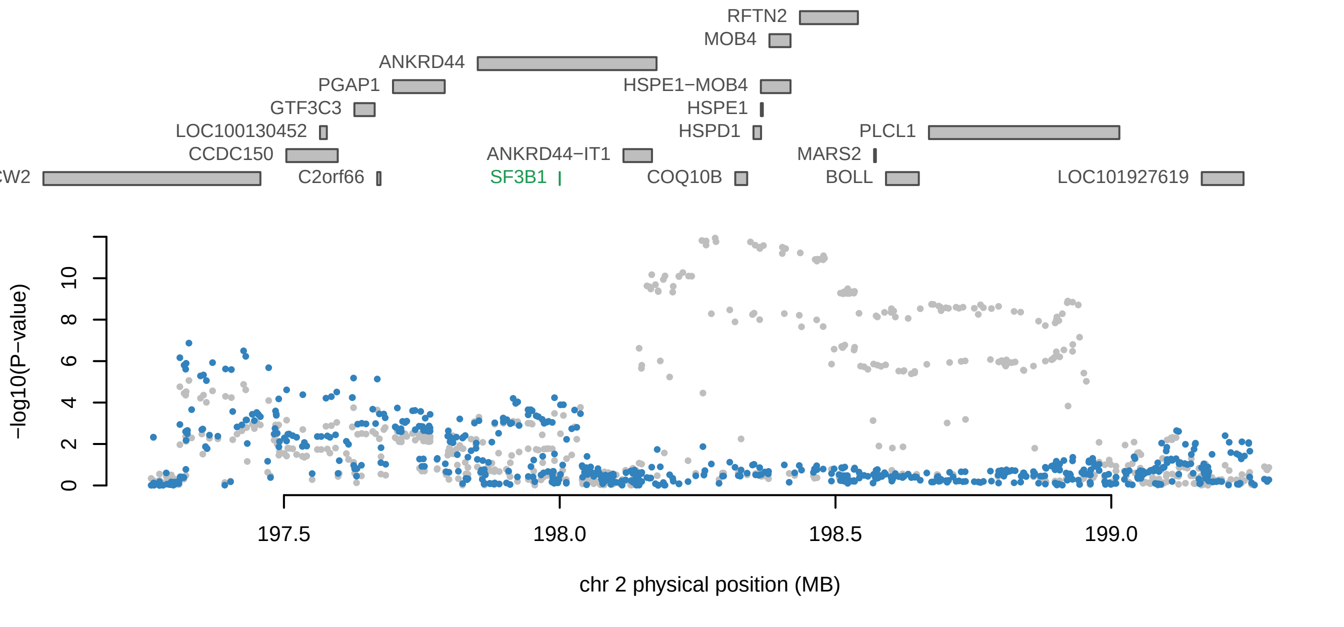
RFTN2
MOB4
ANKRD44
PGAP1
HSPE1−MOB4
GTF3C3
HSPE1
LOC100130452
HSPD1
PLCL1
CCDC150
ANKRD44−IT1
MARS2
W2
C2orf66
SF3B1
COQ10B
BOLL
LOC101927619
−log10(P−value)
0
2
4
6
8
10
197.5
198.0
198.5
199.0
chr 2 physical position (MB)

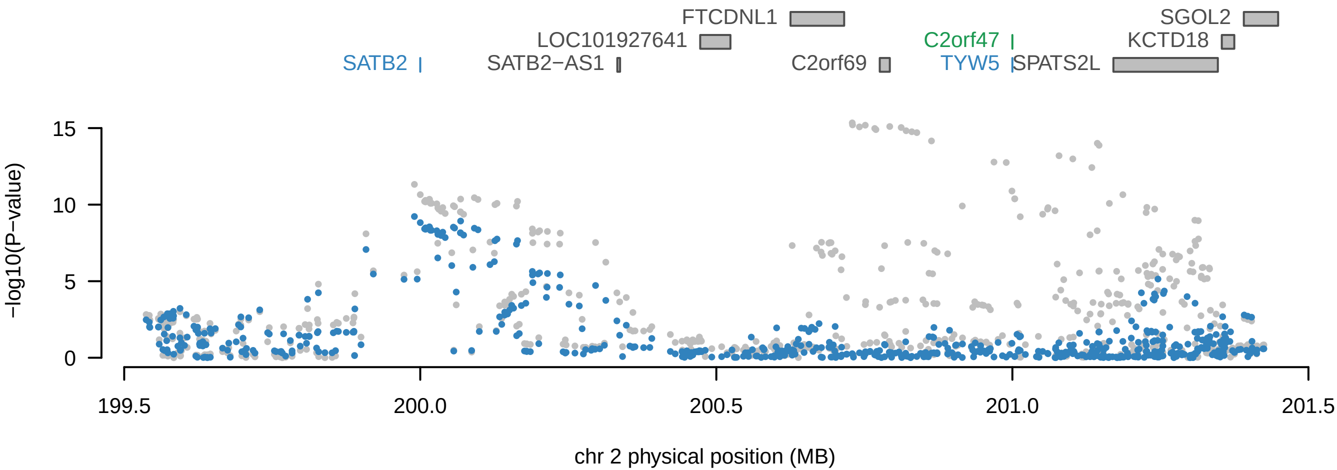
FTCDNL1
SGOL2
LOC101927641
C2orf47
KCTD18
SATB2
SATB2−AS1
C2orf69
TYW5
SPATS2L
15
10
5
0
−log10(P−value)
199.5
200.0
200.5
201.0
201.5
chr 2 physical position (MB)

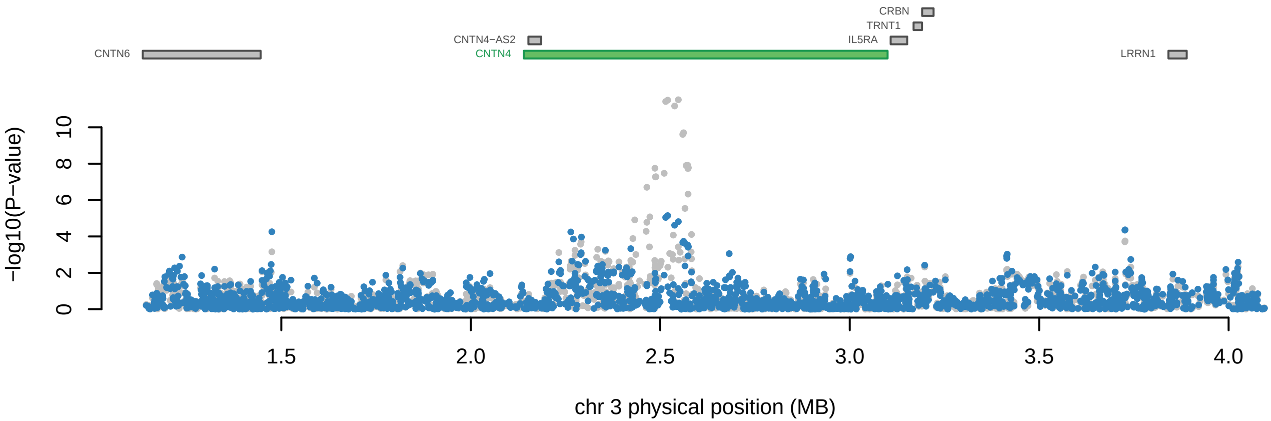
CRBN
TRNT1
CNTN4−AS2
IL5RA
CNTN6
CNTN4
LRRN1
−log10(P−value)
0
2
4
6
8
10
1.5
2.0
2.5
3.0
3.5
4.0
chr 3 physical position (MB)

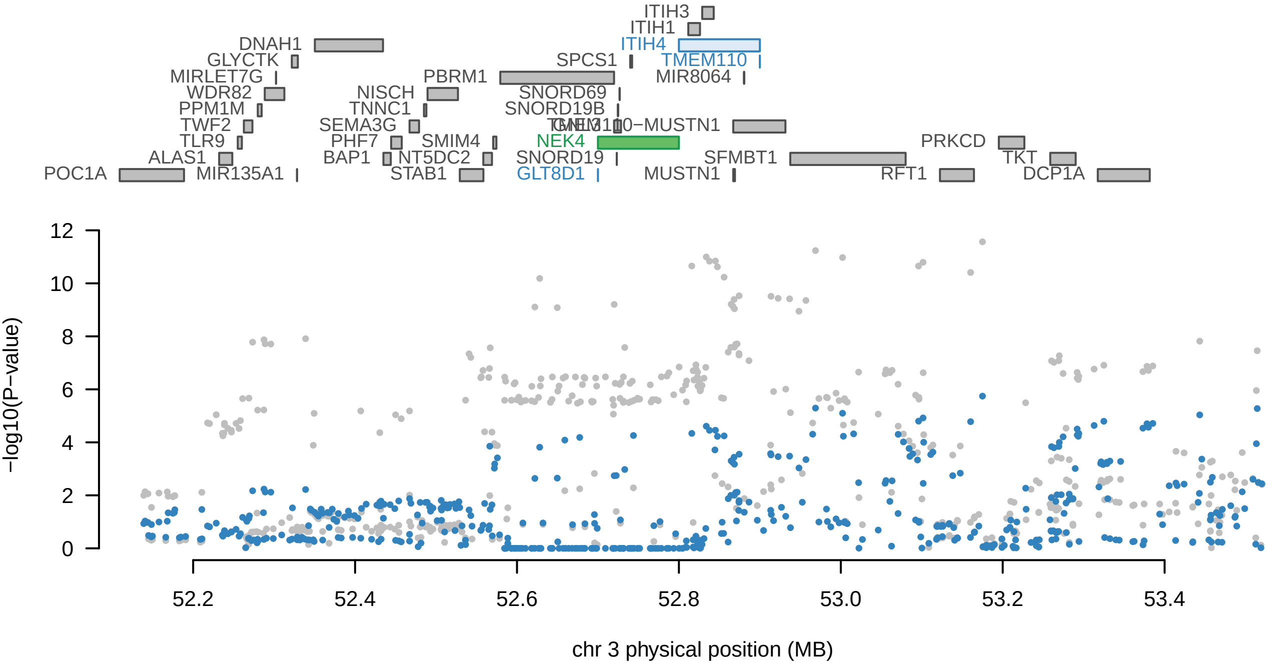
ITIH3
ITIH1
DNAH1
ITIH4
GLYCTK
SPCS1
TMEM110
MIRLET7G
PBRM1
MIR8064
WDR82
NISCH
SNORD69
PPM1M
TNNC1
SNORD19B
TWF2
SEMA3G
TMEM110−MUSTN1
TLR9
PHF7
SMIM4
NEK4
PRKCD
ALAS1
BAP1
NT5DC2
SNORD19
SFMBT1
TKT
POC1A
MIR135A1
STAB1
GLT8D1
MUSTN1
RFT1
DCP1A
−log10(P−value)
0
2
4
6
8
10
12
52.2
52.4
52.6
52.8
53.0
53.2
53.4
chr 3 physical position (MB)

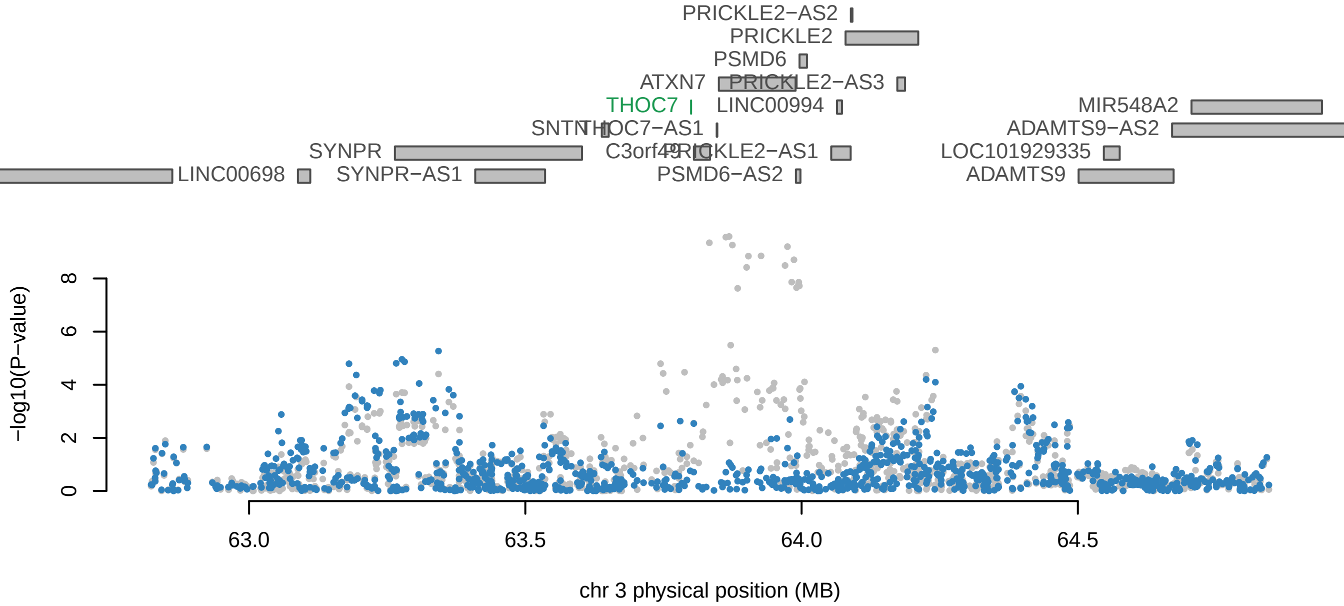
PRICKLE2−AS2
PRICKLE2
PSMD6
ATXN7
PRICKLE2−AS3
THOC7
LINC00994
MIR548A2
SNTN
THOC7−AS1
ADAMTS9−AS2
SYNPR
C3orf49
PRICKLE2−AS1
LOC101929335
LINC00698
SYNPR−AS1
PSMD6−AS2
ADAMTS9
−log10(P−value)
0
2
4
6
8
63.0
63.5
64.0
64.5
chr 3 physical position (MB)

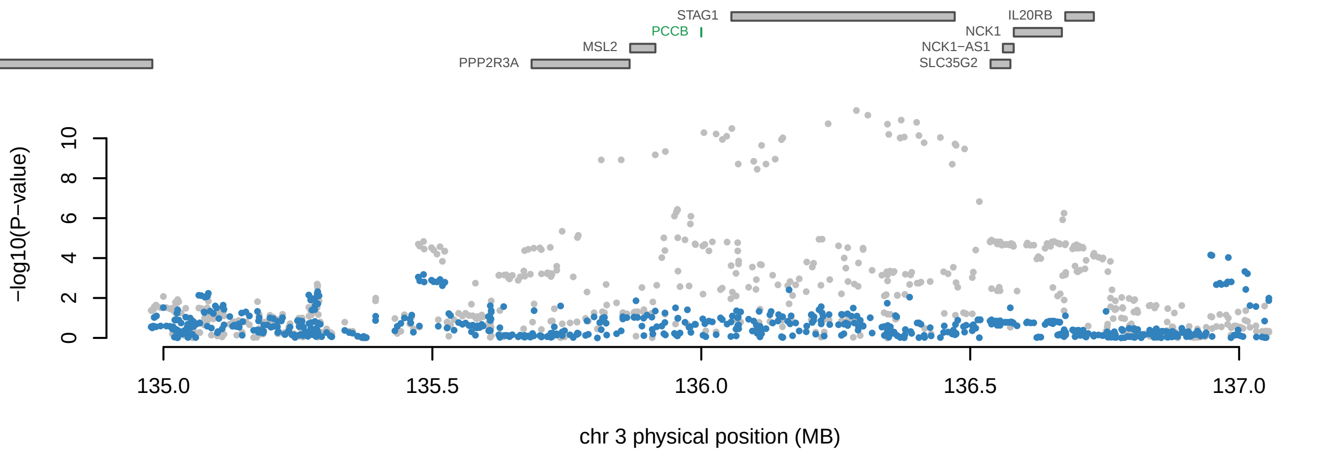

STAG1
IL20RB
PCCB
NCK1
MSL2
NCK1−AS1
PPP2R3A
SLC35G2
−log10(P−value)
0
2
4
6
8
10
135.0
135.5
136.0
136.5
137.0
chr 3 physical position (MB)

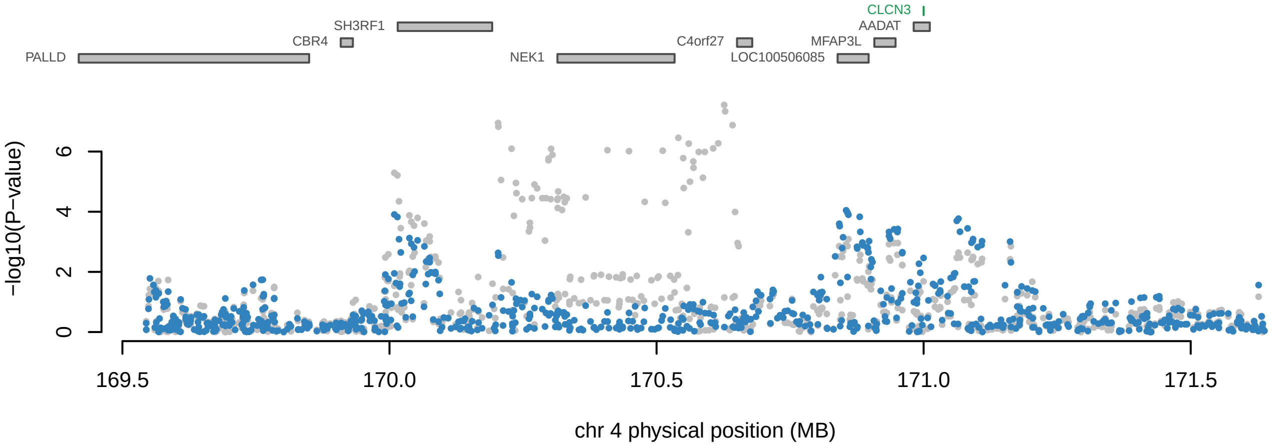
CLCN3
SH3RF1
AADAT
CBR4
C4orf27
MFAP3L
PALLD
NEK1
LOC100506085
−log10(P−value)
0
2
4
6
169.5
170.0
170.5
171.0
171.5
chr 4 physical position (MB)

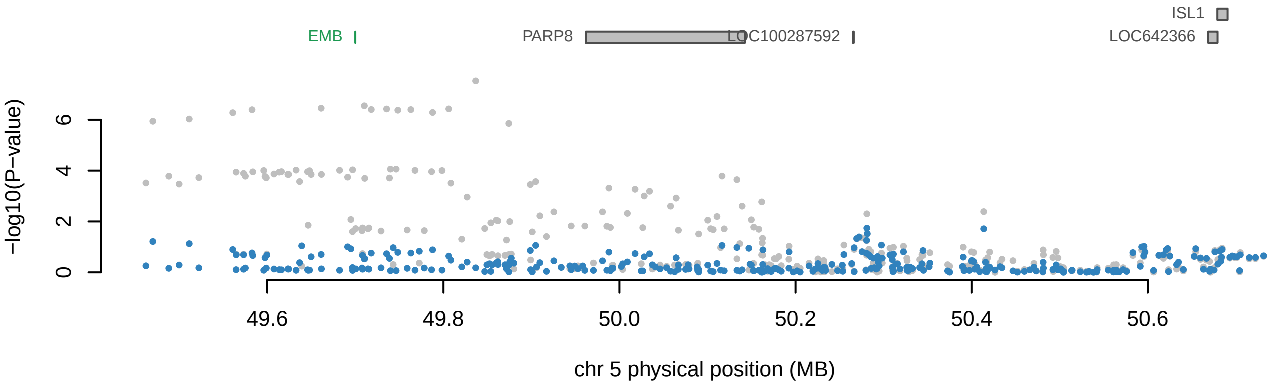

ISL1
EMB
PARP8
LOC100287592
LOC642366
−log10(P−value)
0
2
4
6
49.6
49.8
50.0
50.2
50.4
50.6
chr 5 physical position (MB)

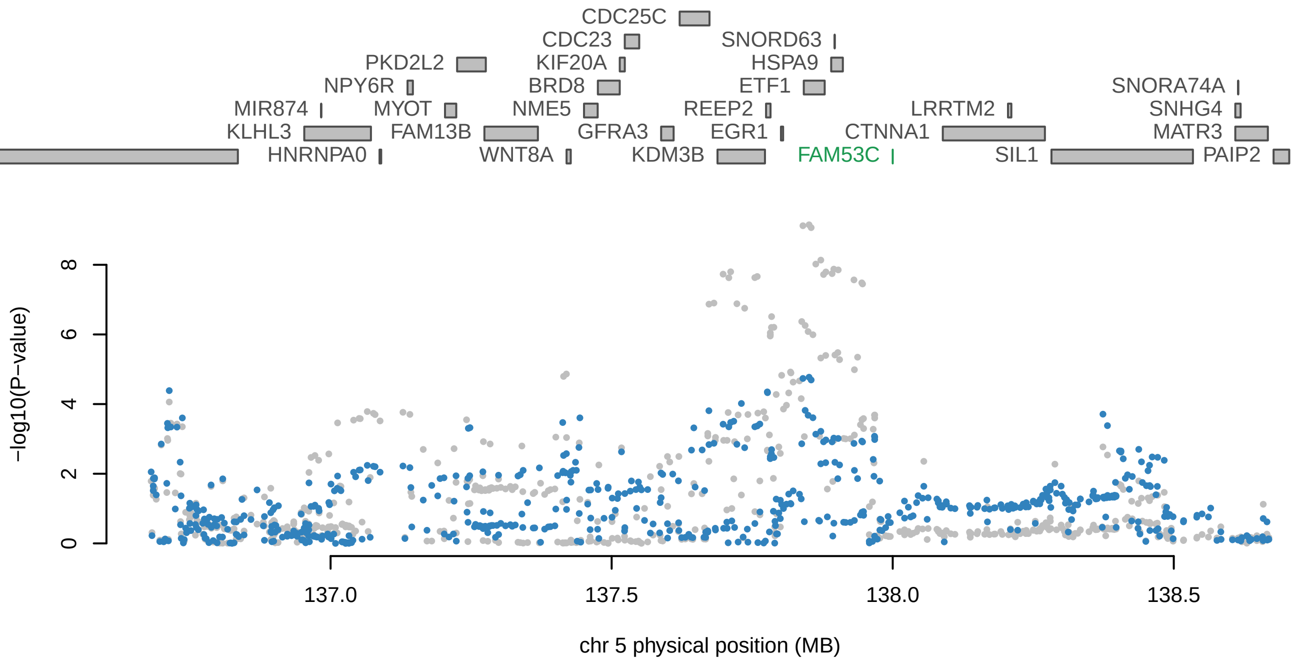
CDC25C
CDC23
SNORD63
PKD2L2
KIF20A
HSPA9
NPY6R
BRD8
ETF1
SNORA74A
MIR874
MYOT
NME5
REEP2
LRRTM2
SNHG4
KLHL3
FAM13B
GFRA3
EGR1
CTNNA1
MATR3
HNRNPA0
WNT8A
KDM3B
FAM53C
SIL1
PAIP2
−log10(P−value)
0
2
4
6
8
137.0
137.5
138.0
138.5
chr 5 physical position (MB)

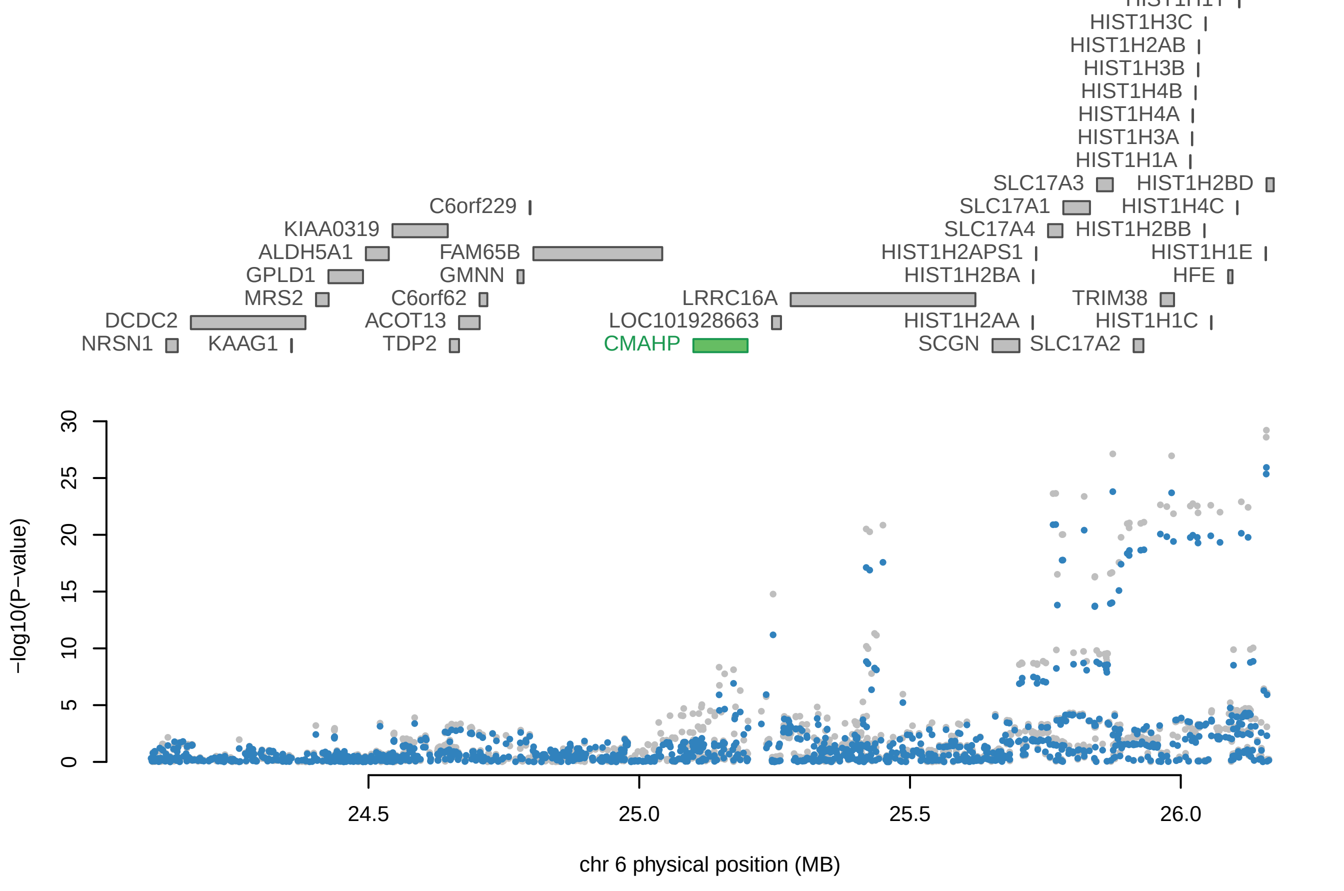

HIST1H3C
HIST1H2AB
HIST1H3B
HIST1H4B
HIST1H4A
HIST1H3A
HIST1H1A
SLC17A3
HIST1H2BD
C6orf229
SLC17A1
HIST1H4C
KIAA0319
SLC17A4
HIST1H2BB
ALDH5A1
FAM65B
HIST1H2APS1
HIST1H1E
GPLD1
GMNN
HIST1H2BA
HFE
MRS2
C6orf62
LRRC16A
TRIM38
DCDC2
ACOT13
LOC101928663
HIST1H2AA
HIST1H1C
NRSN1
KAAG1
TDP2
CMAHP
SCGN
SLC17A2
−log10(P−value)
0
5
10
15
20
25
30
24.5
25.0
25.5
26.0
chr 6 physical position (MB)

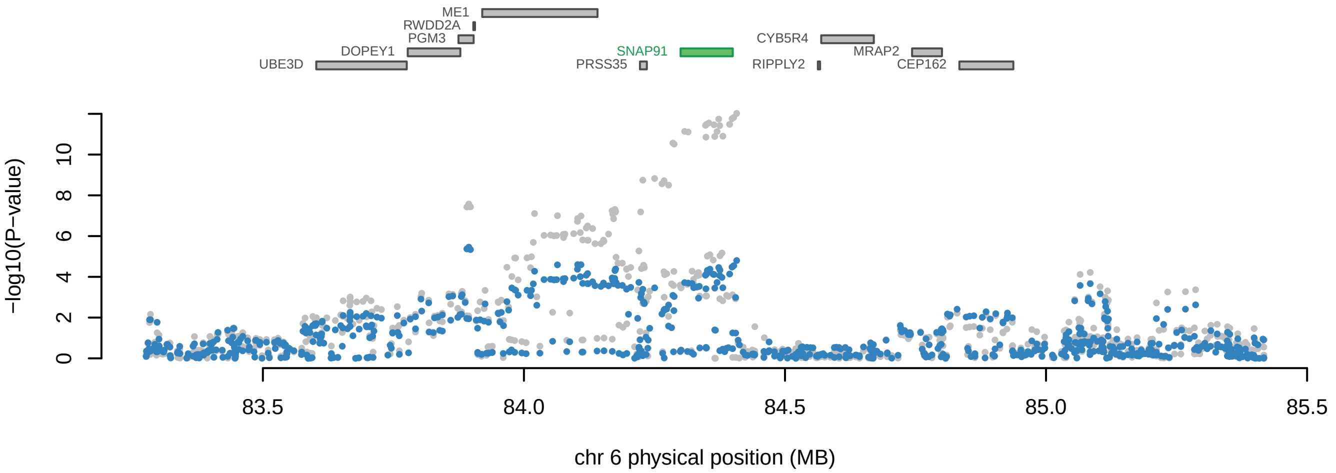

ME1
RWDD2A
PGM3
DOPEY1
UBE3D
SNAP91
PRSS35
CYB5R4
MRAP2
RIPPLY2
CEP162
−log10(P−value)
0
2
4
6
8
10
83.5
84.0
84.5
85.0
85.5
chr 6 physical position (MB)

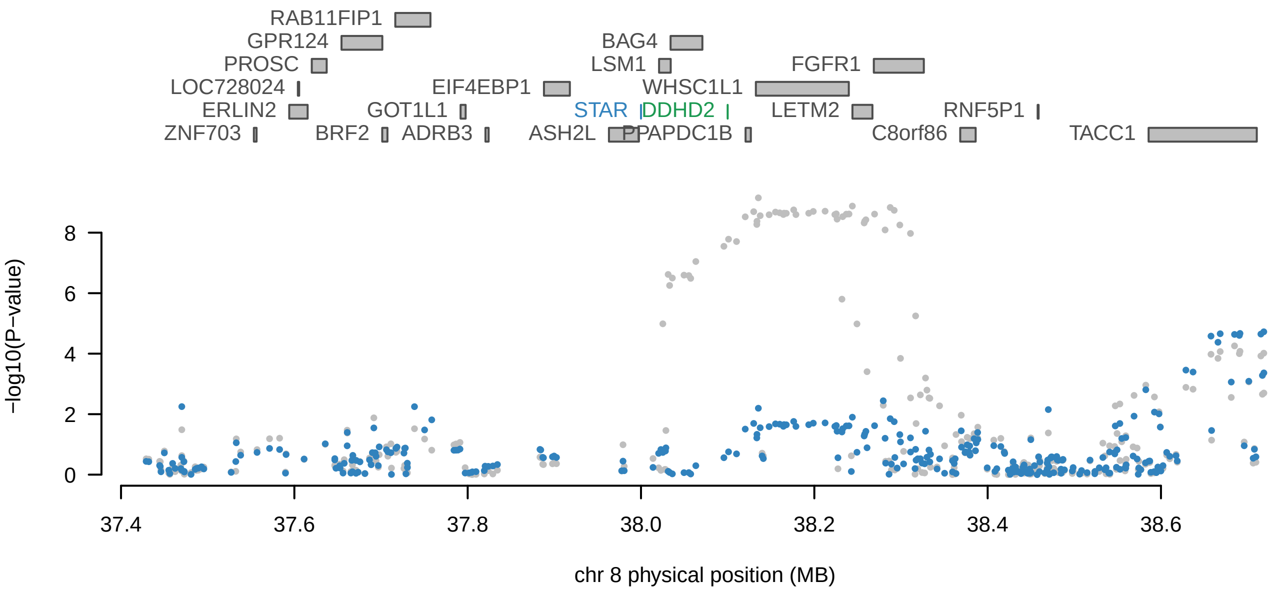
RAB11FIP1
GPR124
BAG4
PROSC
LSM1
FGFR1
LOC728024
EIF4EBP1
WHSC1L1
ERLIN2
GOT1L1
STAR
DDHD2
LETM2
RNF5P1
ZNF703
BRF2
ADRB3
ASH2L
PPAPDC1B
C8orf86
TACC1
−log10(P−value)
0
2
4
6
8
37.4
37.6
37.8
38.0
38.2
38.4
38.6
chr 8 physical position (MB)

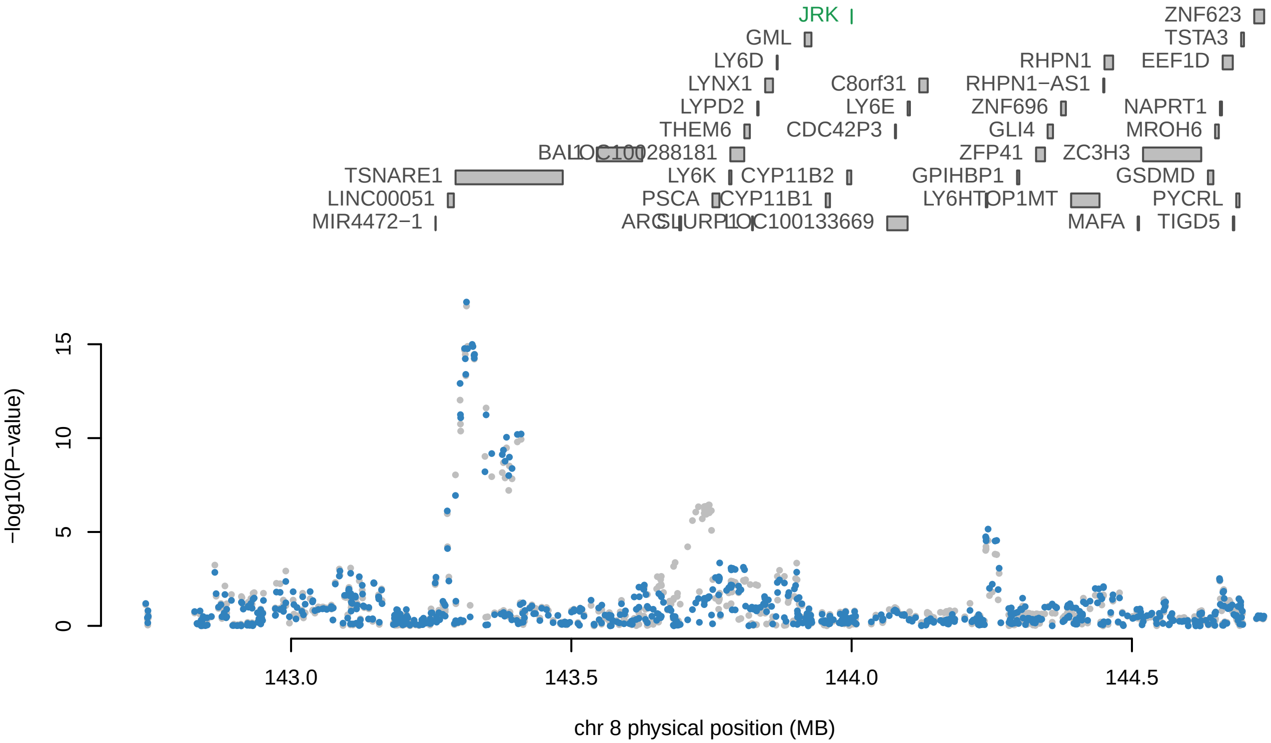

JRK
GML
LY6D
LYNX1
LYPD2
THEM6
BAI1
LOC100288181
TSNARE1
LINC00051
MIR4472−1
LY6K
PSCA
ARC
SLURP1
LOC100133669
CYP11B2
CYP11B1
C8orf31
LY6E
CDC42P3
RHPN1
RHPN1−AS1
ZNF696
GLI4
ZFP41
GPIHBP1
LY6H
TOP1MT
MAFA
ZC3H3
ZNF623
TSTA3
EEF1D
NAPRT1
MROH6
GSDMD
PYCRL
TIGD5
−log10(P−value)
0
5
10
15
143.0
143.5
144.0
144.5
chr 8 physical position (MB)

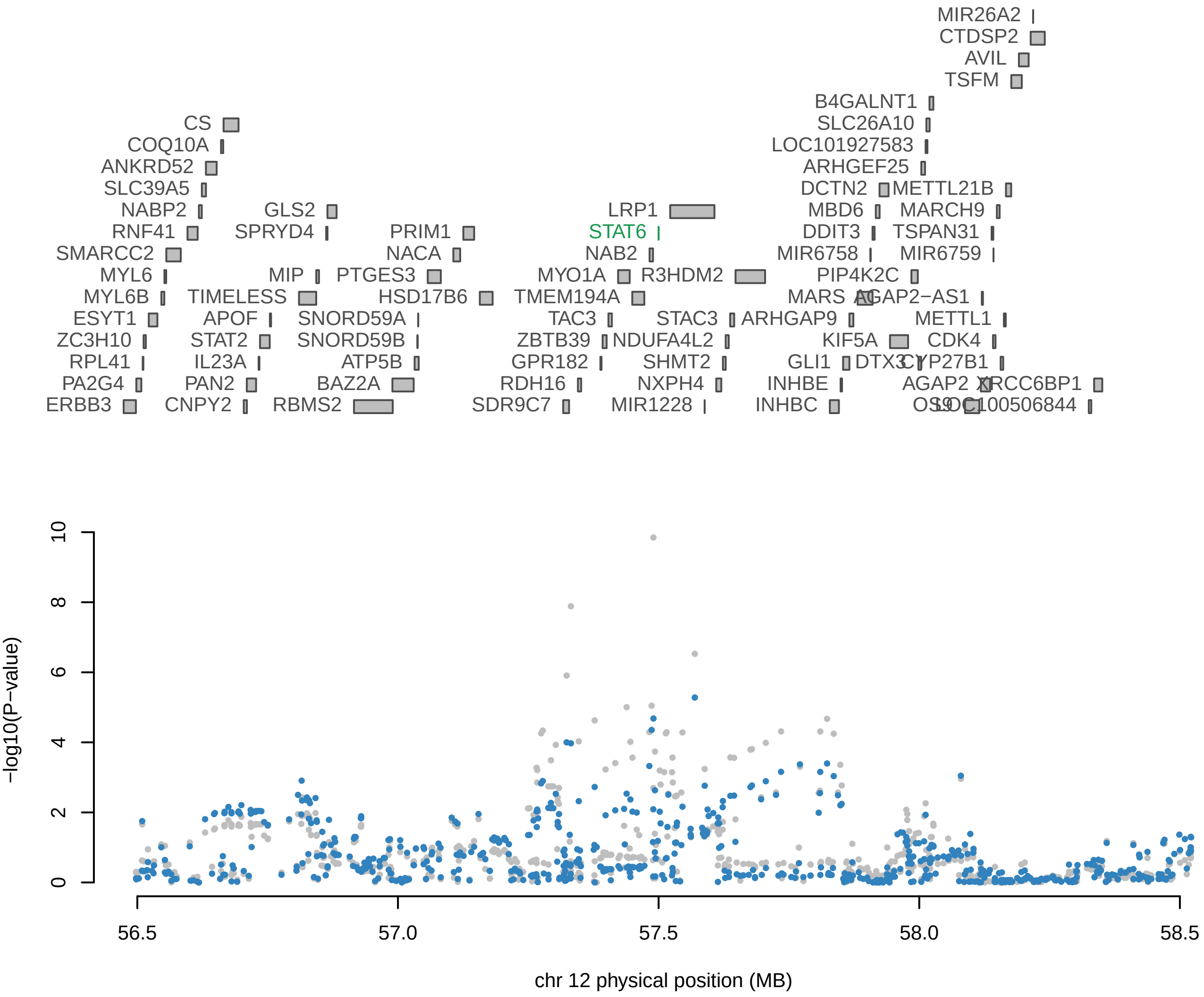

MIR26A2
CTDSP2
AVIL
TSFM
B4GALNT1
SLC26A10
LOC101927583
ARHGEF25
DCTN2
METTL21B
MBD6
MARCH9
DDIT3
TSPAN31
MIR6758
MIR6759
PIP4K2C
MARS
AGAP2−AS1
ARHGAP9
METTL1
KIF5A
CDK4
GLI1
DTX3
CYP27B1
INHBE
AGAP2
XRCC6BP1
INHBC
OS9
LOC100506844
CS
COQ10A
ANKRD52
SLC39A5
NABP2
GLS2
RNF41
SPRYD4
PRIM1
SMARCC2
NACA
MYL6
MIP
PTGES3
MYL6B
TIMELESS
HSD17B6
ESYT1
APOF
SNORD59A
ZC3H10
STAT2
SNORD59B
RPL41
IL23A
ATP5B
PA2G4
PAN2
BAZ2A
ERBB3
CNPY2
RBMS2
LRP1
STAT6
NAB2
MYO1A
R3HDM2
TMEM194A
TAC3
STAC3
ZBTB39
NDUFA4L2
GPR182
SHMT2
RDH16
NXPH4
SDR9C7
MIR1228
−log10(P−value)
0
2
4
6
8
10
56.5
57.0
57.5
58.0
58.5
chr 12 physical position (MB)

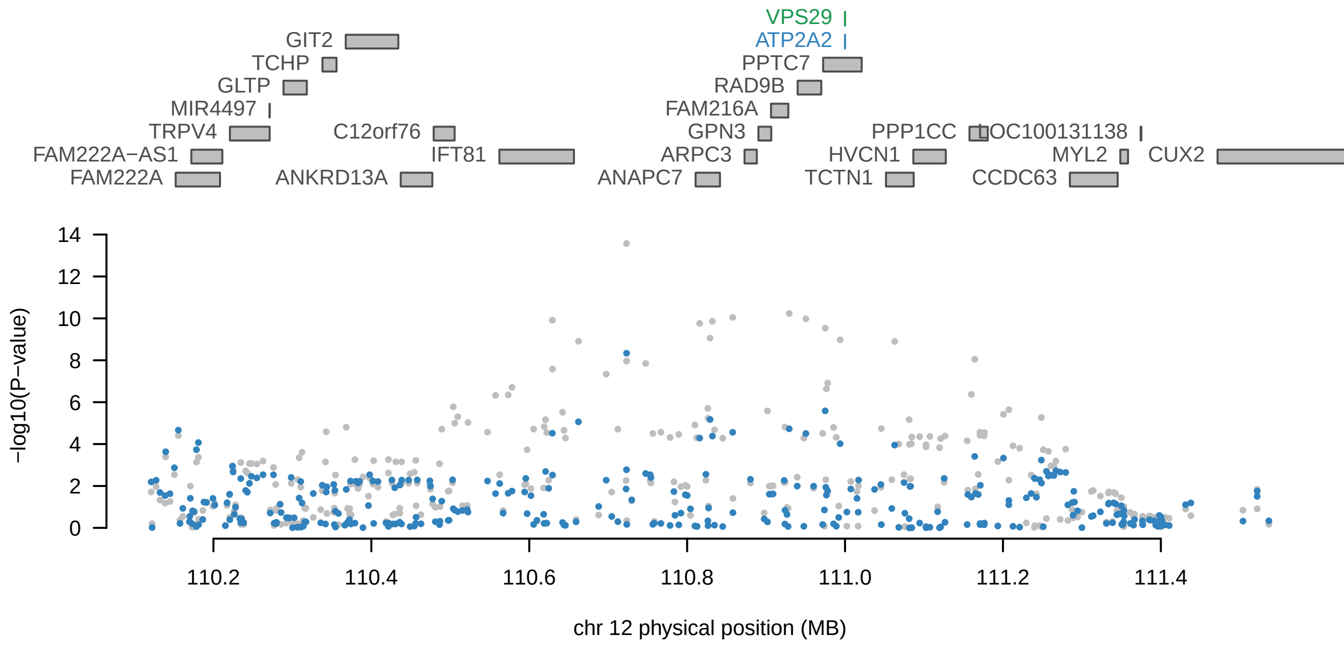

VPS29
ATP2A2
GIT2
TCHP
PPTC7
GLTP
RAD9B
MIR4497
FAM216A
TRPV4
C12orf76
GPN3
PPP1CC
LOC100131138
FAM222A−AS1
IFT81
ARPC3
HVCN1
MYL2
CUX2
FAM222A
ANKRD13A
ANAPC7
TCTN1
CCDC63
−log10(P−value)
0
2
4
6
8
10
12
14
110.2
110.4
110.6
110.8
111.0
111.2
111.4
chr 12 physical position (MB)

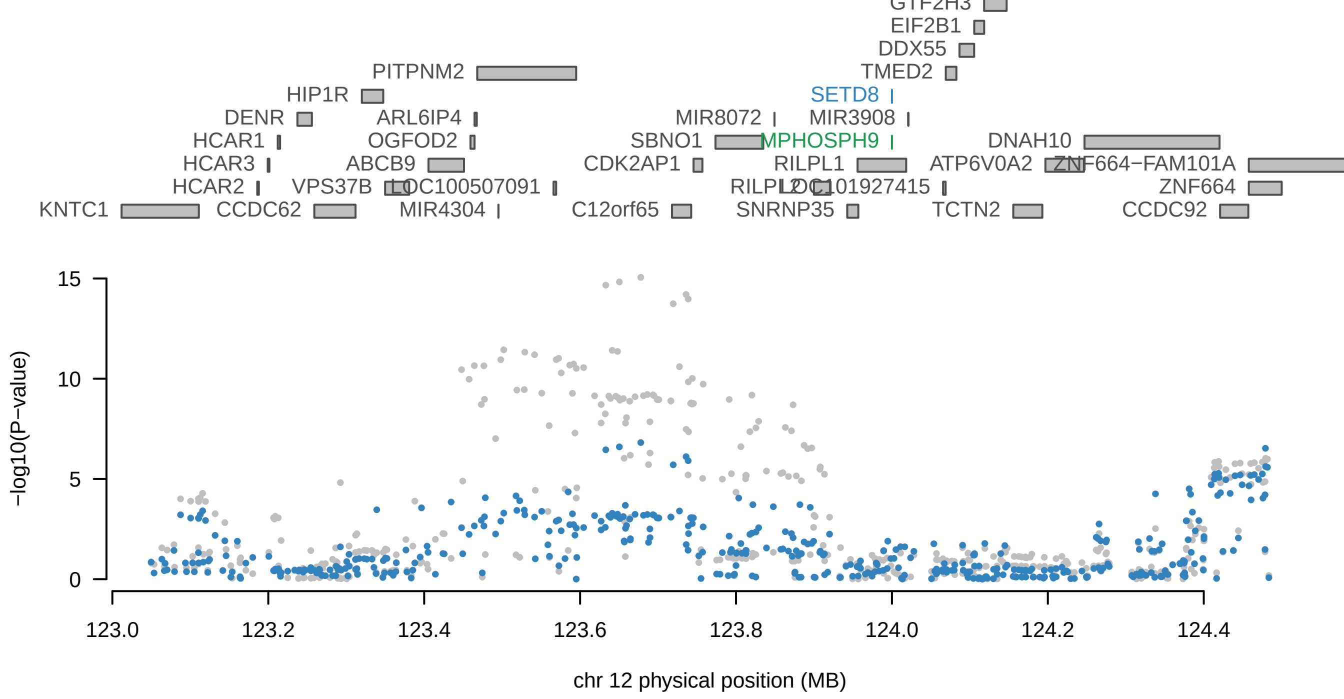
GTF2H3
EIF2B1
DDX55
PITPNM2
TMED2
HIP1R
SETD8
DENR
ARL6IP4
MIR8072
MIR3908
HCAR1
OGFOD2
SBNO1
MPHOSPH9
DNAH10
HCAR3
ABCB9
CDK2AP1
RILPL1
ATP6V0A2
ZNF664−FAM101A
HCAR2
VPS37B
LOC100507091
RILPL2
LOC101927415
ZNF664
KNTC1
CCDC62
MIR4304
C12orf65
SNRNP35
TCTN2
CCDC92
−log10(P−value)
15
10
5
0
123.0
123.2
123.4
123.6
123.8
124.0
124.2
124.4
chr 12 physical position (MB)

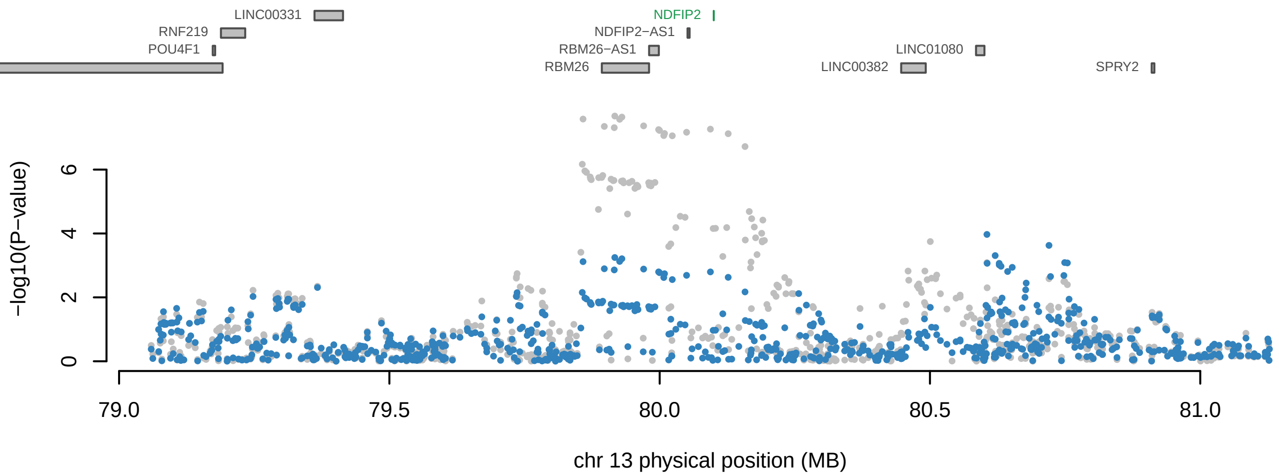

LINC00331
RNF219
POU4F1
NDFIP2
NDFIP2−AS1
RBM26−AS1
RBM26
LINC01080
LINC00382
SPRY2
−log10(P−value)
0
2
4
6
79.0
79.5
80.0
80.5
81.0
chr 13 physical position (MB)

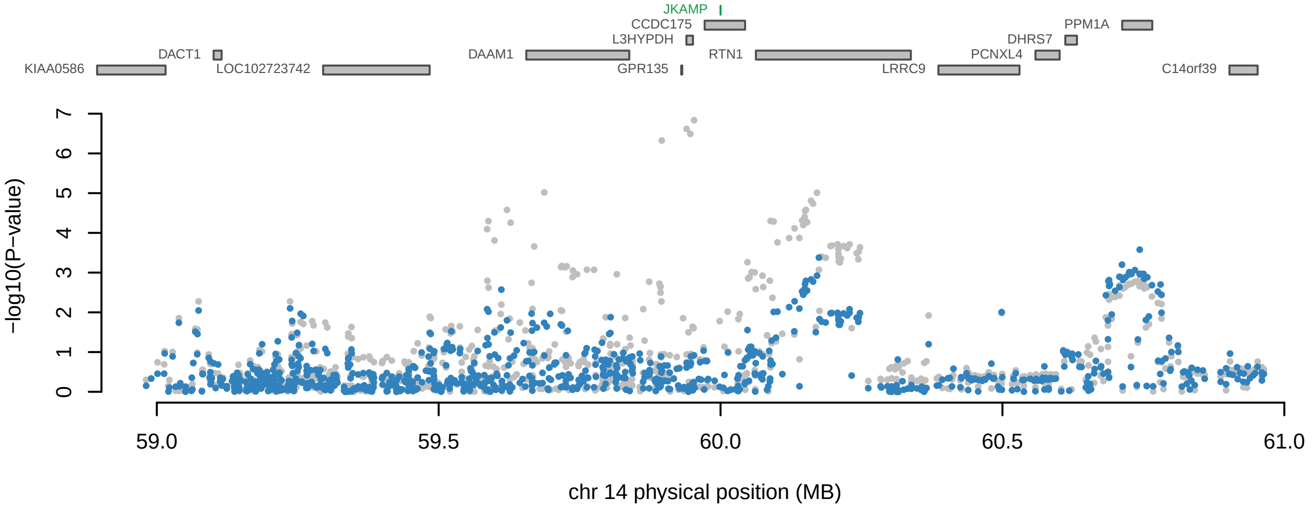

JKAMP
CCDC175
PPM1A
L3HYPDH
DHRS7
DACT1
DAAM1
RTN1
PCNXL4
KIAA0586
LOC102723742
GPR135
LRRC9
C14orf39
−log10(P−value)
0
1
2
3
4
5
6
7
59.0
59.5
60.0
60.5
61.0
chr 14 physical position (MB)

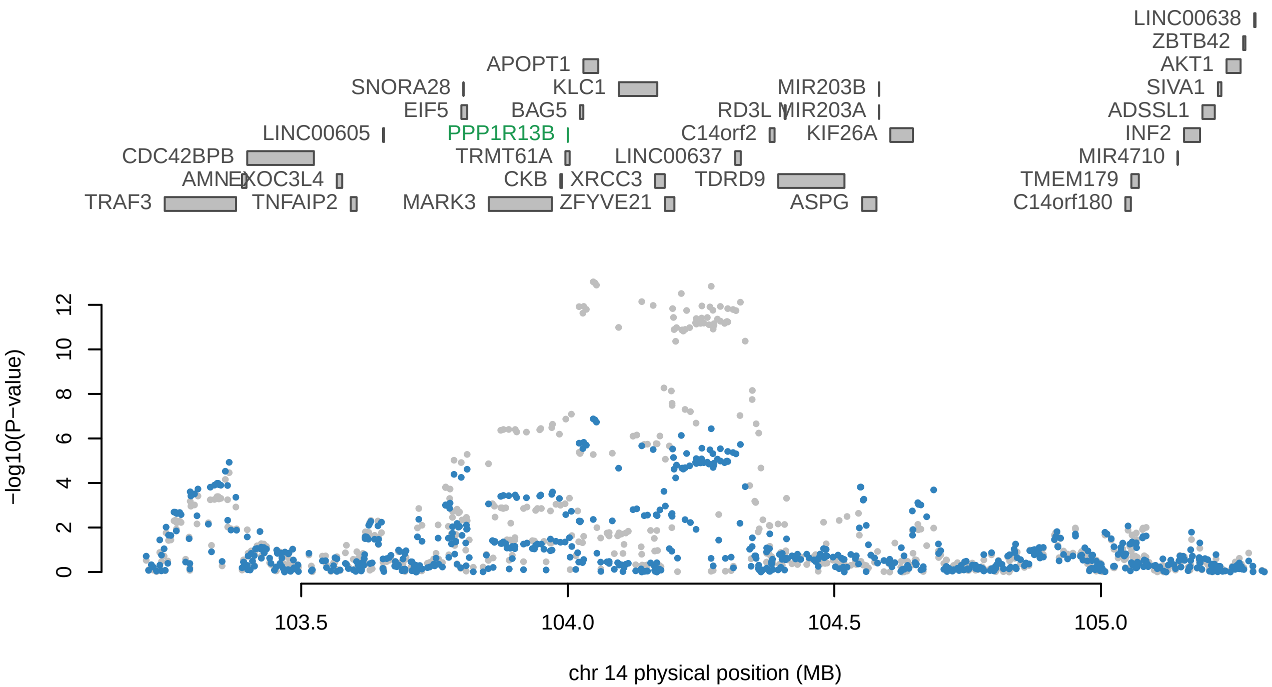
LINC00638
ZBTB42
APOPT1
AKT1
SNORA28
KLC1
MIR203B
SIVA1
EIF5
BAG5
RD3L
MIR203A
ADSSL1
LINC00605
PPP1R13B
C14orf2
KIF26A
INF2
CDC42BPB
TRMT61A
LINC00637
MIR4710
AMN
EXOC3L4
CKB
XRCC3
TDRD9
TMEM179
TRAF3
TNFAIP2
MARK3
ZFYVE21
ASPG
C14orf180
−log10(P−value)
0
2
4
6
8
10
12
103.5
104.0
104.5
105.0
chr 14 physical position (MB)

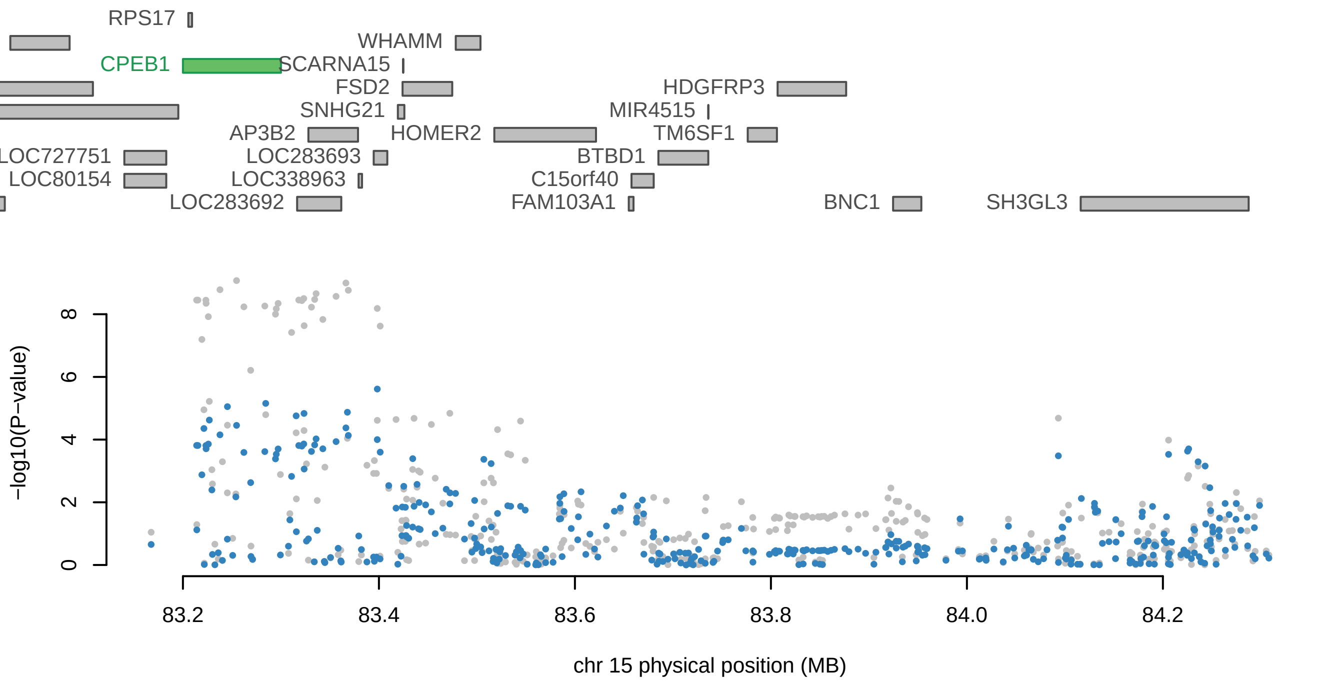

RPS17
WHAMM
CPEB1
SCARNA15
FSD2
HDGFRP3
SNHG21
MIR4515
AP3B2
HOMER2
TM6SF1
LOC727751
LOC283693
BTBD1
LOC80154
LOC338963
C15orf40
LOC283692
FAM103A1
BNC1
SH3GL3
−log10(P−value)
0
2
4
6
8
83.2
83.4
83.6
83.8
84.0
84.2
chr 15 physical position (MB)

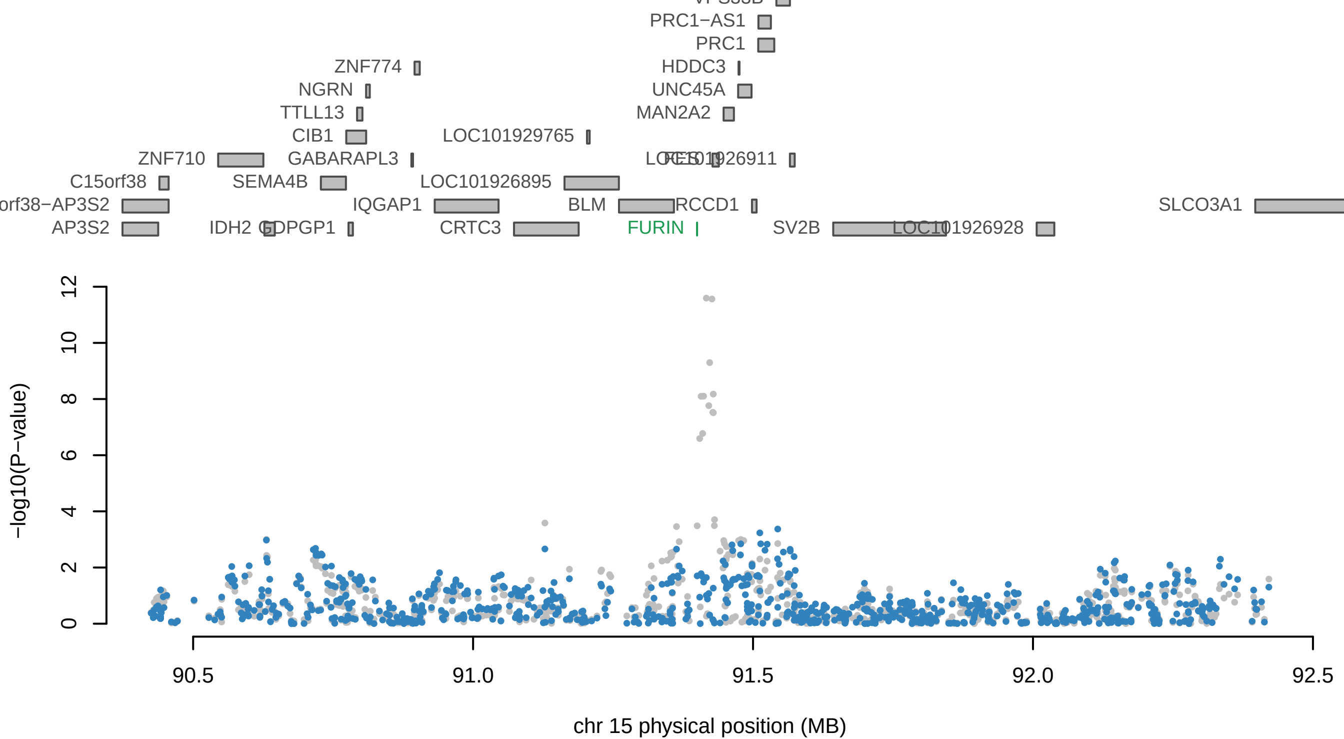

PRC1−AS1
PRC1
ZNF774
HDDC3
NGRN
UNC45A
TTLL13
MAN2A2
CIB1
LOC101929765
ZNF710
GABARAPL3
LOC101926911
C15orf38
SEMA4B
LOC101926895
IQGAP1
BLM
RCCD1
SLCO3A1
AP3S2
IDH2
CRTC3
FURIN
SV2B
LOC101926928
−log10(P−value)
0
2
4
6
8
10
12
90.5
91.0
91.5
92.0
92.5
chr 15 physical position (MB)

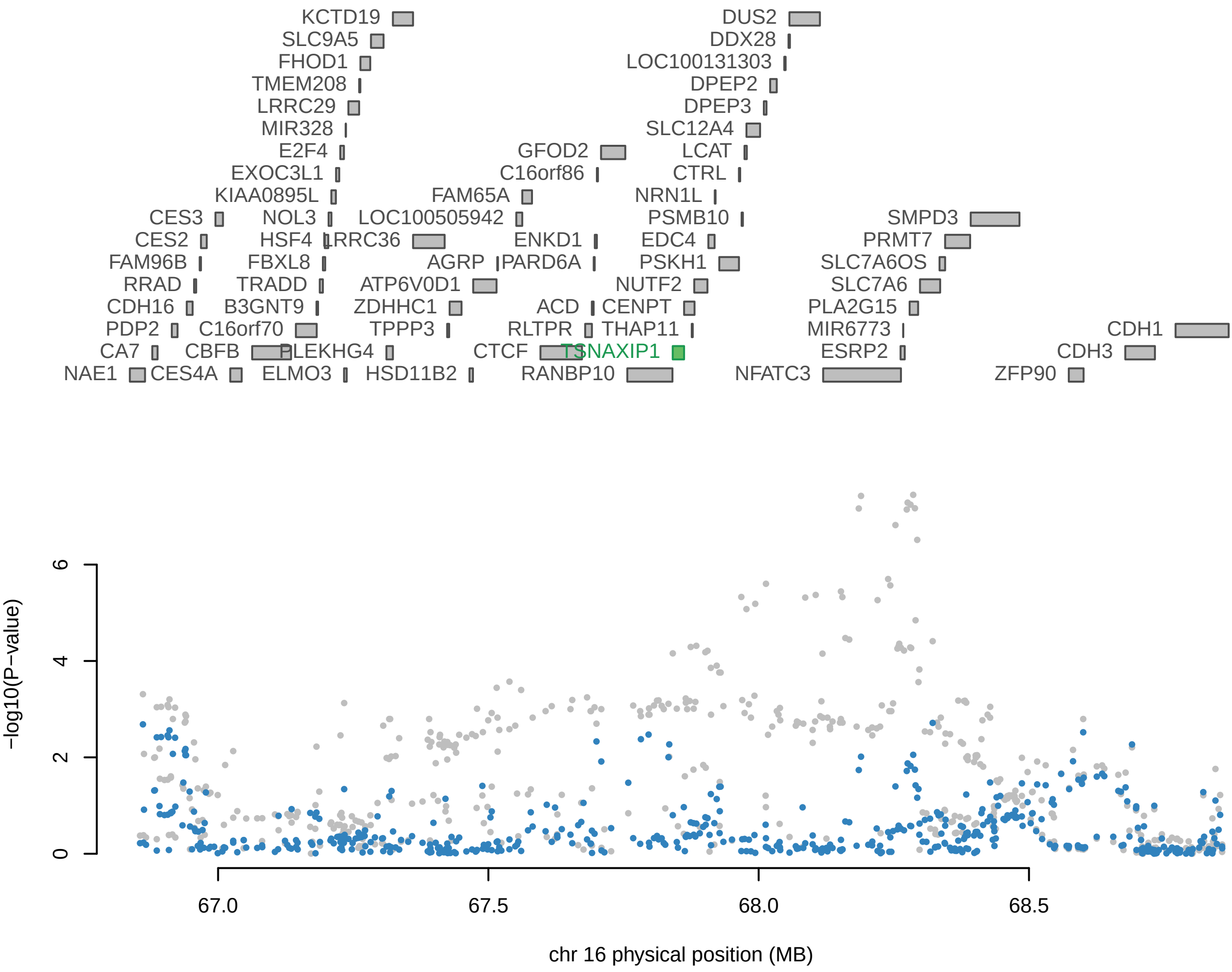

KCTD19
SLC9A5
FHOD1
TMEM208
LRRC29
MIR328
E2F4
EXOC3L1
KIAA0895L
CES3
NOL3
HSF4
LRRC36
CES2
FAM96B
FBXL8
RRAD
TRADD
CDH16
B3GNT9
PDP2
C16orf70
CA7
CBFB
PLEKHG4
NAE1
CES4A
ELMO3
HSD11B2
GFOD2
C16orf86
FAM65A
LOC100505942
ENKD1
AGRP
PARD6A
ATP6V0D1
ZDHHC1
ACD
TPPP3
RLTPR
CTCF
TSNAXIP1
RANBP10
CENPT
THAP11
DUS2
DDX28
LOC100131303
DPEP2
DPEP3
SLC12A4
LCAT
CTRL
NRN1L
PSMB10
EDC4
PSKH1
NUTF2
NFATC3
SMPD3
PRMT7
SLC7A6OS
SLC7A6
PLA2G15
MIR6773
ESRP2
CDH1
CDH3
ZFP90
−log10(P−value)
0
2
4
6
67.0
67.5
68.0
68.5
chr 16 physical position (MB)

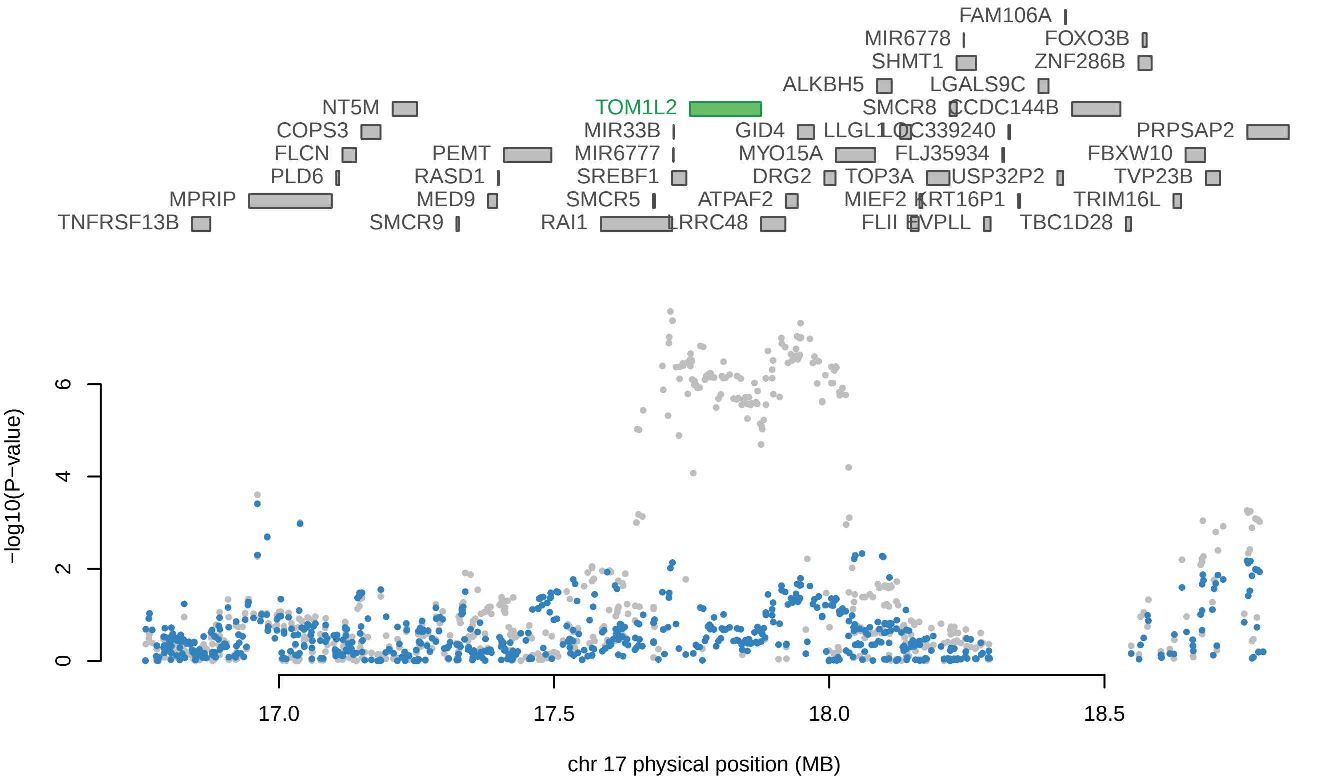

FAM106A
MIR6778
FOXO3B
SHMT1
ZNF286B
ALKBH5
LGALS9C
NT5M
TOM1L2
SMCR8
CCDC144B
COPS3
MIR33B
GID4
LLGL1
LOC339240
PRPSAP2
FLCN
PEMT
MIR6777
MYO15A
FLJ35934
FBXW10
PLD6
RASD1
SREBF1
DRG2
TOP3A
USP32P2
TVP23B
MPRIP
MED9
SMCR5
ATPAF2
MIEF2
KRT16P1
TRIM16L
TNFRSF13B
SMCR9
RAI1
LRRC48
FLII
EVPLL
TBC1D28
−log10(P−value)
0
2
4
6
17.0
17.5
18.0
18.5
chr 17 physical position (MB)

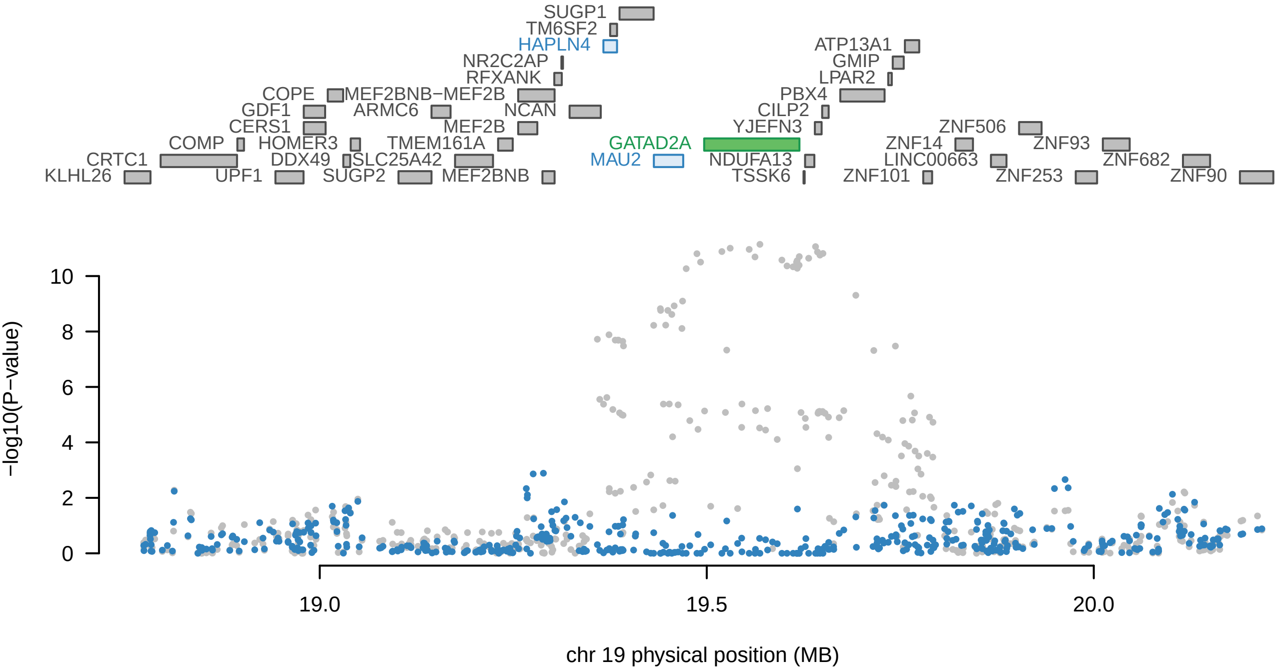

SUGP1
TM6SF2
HAPLN4
NR2C2AP
RFXANK
COPE
MEF2BNB−MEF2B
GDF1
ARMC6
NCAN
CERS1
MEF2B
COMP
HOMER3
TMEM161A
CRTC1
DDX49
SLC25A42
KLHL26
UPF1
SUGP2
MEF2BNB
GATAD2A
MAU2
ATP13A1
GMIP
LPAR2
PBX4
CILP2
YJEFN3
NDUFA13
TSSK6
ZNF506
ZNF14
ZNF93
LINC00663
ZNF682
ZNF101
ZNF253
ZNF90
−log10(P−value)
0
2
4
6
8
10
19.0
19.5
20.0
chr 19 physical position (MB)

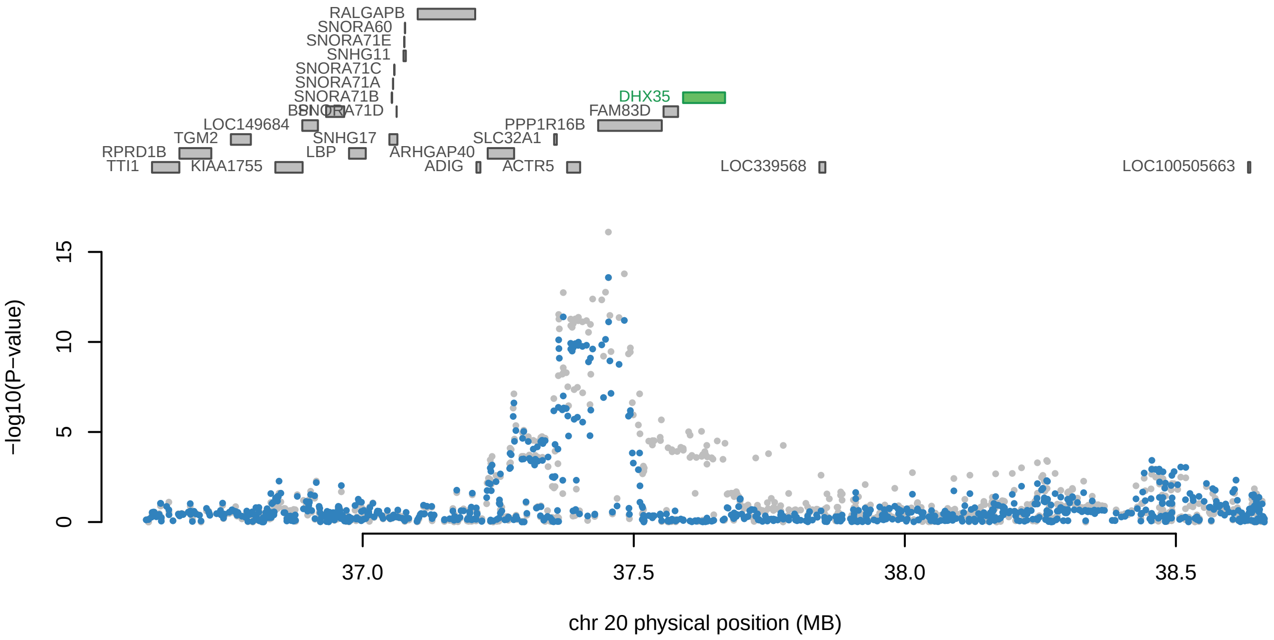
RALGAPB
SNORA60
SNORA71E
SNHG11
SNORA71C
SNORA71A
SNORA71B
DHX35
SNORA71D
FAM83D
LOC149684
PPP1R16B
TGM2
SNHG17
SLC32A1
RPRD1B
LBP
ARHGAP40
TTI1
KIAA1755
ADIG
ACTR5
LOC339568
LOC100505663
−log10(P−value)
0
5
10
15
37.0
37.5
38.0
38.5
chr 20 physical position (MB)

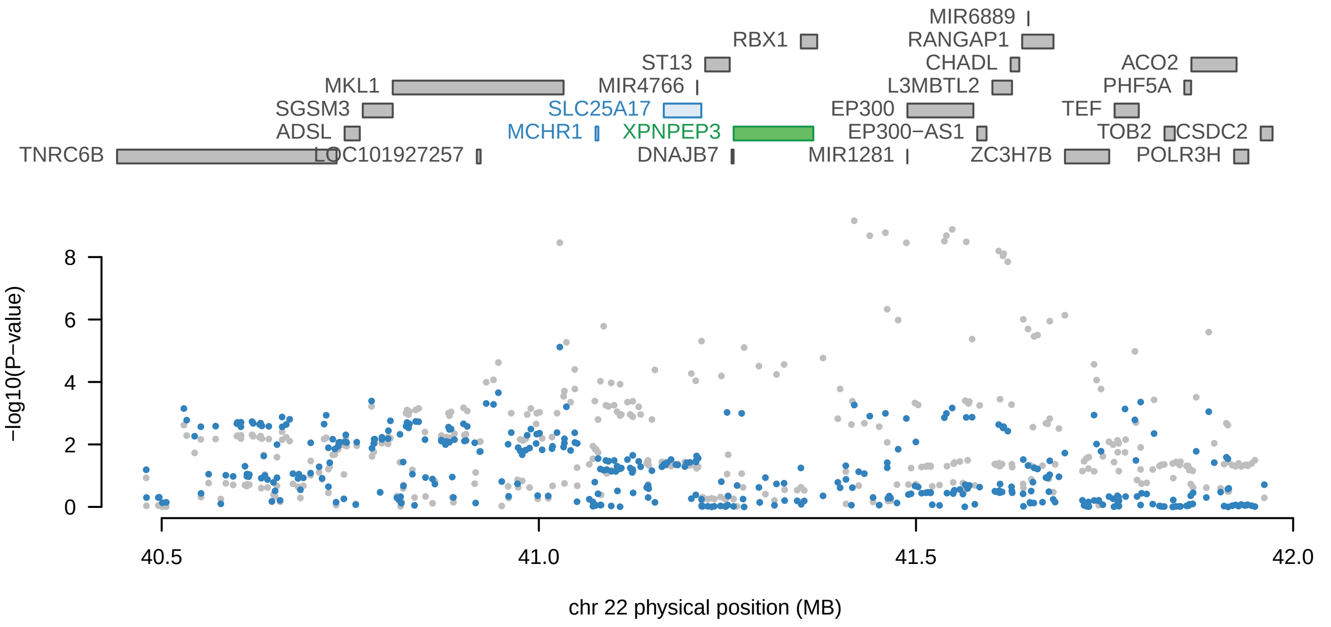

MIR6889
RBX1
RANGAP1
ST13
CHADL
ACO2
MKL1
MIR4766
L3MBTL2
PHF5A
SGSM3
SLC25A17
EP300
TEF
ADSL
MCHR1
XPNPEP3
EP300−AS1
TOB2
CSDC2
TNRC6B
LOC101927257
DNAJB7
MIR1281
ZC3H7B
POLR3H
8
6
4
2
0
−log10(P−value)
40.5
41.0
41.5
42.0
chr 22 physical position (MB)

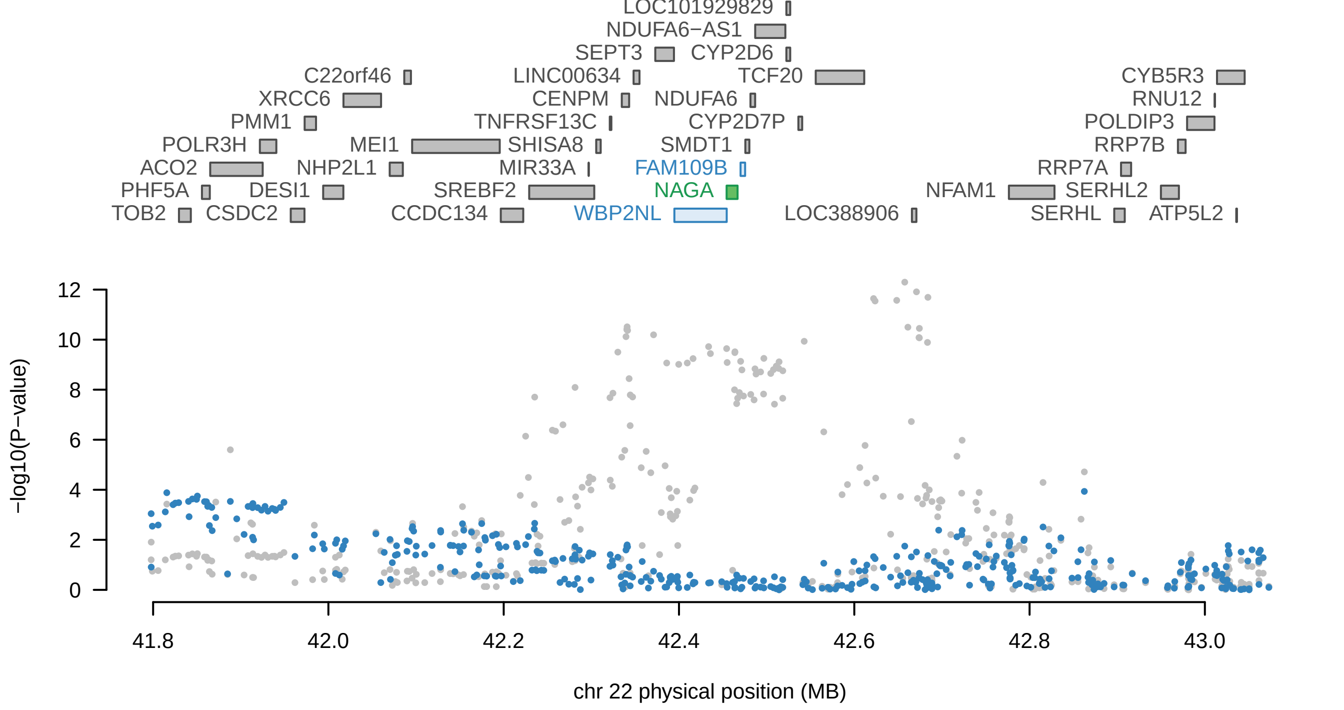
LOC101929829
NDUFA6−AS1
SEPT3
CYP2D6
C22orf46
LINC00634
TCF20
CYB5R3
XRCC6
CENPM
NDUFA6
RNU12
PMM1
TNFRSF13C
CYP2D7P
POLDIP3
POLR3H
MEI1
SHISA8
SMDT1
RRP7B
ACO2
NHP2L1
MIR33A
FAM109B
RRP7A
PHF5A
DESI1
SREBF2
NAGA
NFAM1
SERHL2
TOB2
CSDC2
CCDC134
WBP2NL
LOC388906
SERHL
ATP5L2
−log10(P−value)
0
2
4
6
8
10
12
41.8
42.0
42.2
42.4
42.6
42.8
43.0
chr 22 physical position (MB)
